# Energy Transfer in Mixed Convection MHD Flow of Nanofluid Containing Different Shapes of Nanoparticles in a Channel Filled with Saturated Porous Medium

**DOI:** 10.1186/s11671-015-1144-4

**Published:** 2015-12-23

**Authors:** Gul Aaiza, Ilyas Khan, Sharidan Shafie

**Affiliations:** Department of Mathematical Sciences, Faculty of Science, Universiti Teknologi, 81310, UTM, Skudai, Malaysia; Basic Engineering Sciences Department, College of of Engineering Majmaah University, Majmaah, 11952 Saudi Arabia

**Keywords:** Mixed convection, Nanofluid, Heat transfer, Cylindrical shaped nanoparticles, MHD flow, Porous medium, Analytical solutions

## Abstract

Energy transfer in mixed convection unsteady magnetohydrodynamic (MHD) flow of an incompressible nanofluid inside a channel filled with saturated porous medium is investigated. The channel with non-uniform walls temperature is taken in a vertical direction under the influence of a transverse magnetic field. Based on the physical boundary conditions, three different flow situations are discussed. The problem is modelled in terms of partial differential equations with physical boundary conditions. Four different shapes of nanoparticles of equal volume fraction are used in conventional base fluids, ethylene glycol (EG) (*C*_*2*_*H*_*6*_*O*_*2*_) and water (*H*_*2*_*O*). Solutions for velocity and temperature are obtained discussed graphically in various plots. It is found that viscosity and thermal conductivity are the most prominent parameters responsible for different results of velocity and temperature. Due to higher viscosity and thermal conductivity, *C*_*2*_*H*_*6*_*O*_*2*_ is regarded as better convectional base fluid compared to *H*_*2*_*O*.

## Introduction

Thermal conductivity plays a vital role in heat transfer enhancement. Conventional heat transfer fluids such as water, ethylene glycol (EG), kerosene oil and lubricant oils have poor thermal conductivities compared to solids. Solids particles on the other hand have higher thermal conductivities compared to conventional heat transfer fluids. Choi [1] in his pioneering work indicated that when a small amount of nanoparticles is added to common base fluids, it increases significantly the thermal conductivity of the base fluids as well as their convective heat transfer rate. This mixtures is known as nanofluids. More exactly, nanofluids are suspensions of nano-size particles in base fluids. Usually nanofluids contain different types of nanoparticles such as oxides, metals and carbides in commonly base fluids like water, EG, propylene glycol and kerosene oil. Some specific applications of nanofluids are found in various electronic equipment, energy supply, power generation, air conditioning and production. Vajjha and Das [2] for the first time used EG (60 %) and water (40 %) mixture as base fluid for the preparation of alumina (*Al*_*2*_*O*_*3*_), copper oxide (*CuO*) and zinc oxide (*ZnO*)nanofluids. At the same temperature and concentration, they found that *CuO* nanofluid posses high thermal conductivity compare to those of *Al*_*2*_*O*_*3*_ and *ZnO* nanofluids. Naik and Sundar [3] took 70 % propylene glycol and 30 % water and prepared *CuO* nanofluid. As expected, they found that *CuO* nanofluid has better thermal conductivity and viscosity properties compare to base fluid. Recently, Mansur et al. [4] studied nanofluids for magnetohydrodynamic (MHD) stagnation point flow past a permeable sheet for stretching and shrinking cases. They obtained numerical solutions using bvp4c program in MATLAB and computed results for embedded parameters.

The ability of nanoparticles to enhance the thermal conductivity of base fluids together with numerous applications of nanofluids in industry has attracted the interest of researchers to conduct further studies. Amongst them, several are performing experimental work, some of them are using numerical computations, however very few studies are available on analytic side. Perhaps, it is due to the reason that analytic solutions are not always convenient. Among the various attempts are mention here those made in [5–15].

The quality of nanofluid not only depends on the type of nanoparticles but also their shapes. Researchers usually use nanoparticles of spherical shapes. However, in terms of applications and significance, spherical shaped nanoparticles are limited. Due to this reason non-spherical shaped nanoparticles are choose in this study. More exactly, this study incorporates four different types of nanoparticles namely cylinder, platelet, blade and brick. Furthermore, nanofluids literature reveals that non-spherical shaped nanoparticles carry a number of key desirable properties to be the main focus of current research especially in cancer therapy. Recently, investigation shows that cylindrical shaped nanoparticles are seven times more deadly than traditional spherical shaped nanoparticles in the delivery of drug to breast cancer cells. To the best of author knowledge, analytic studies on different shapes of nanoparticles contained in EG or water as the base fluids is not reported yet. Although, Timofeeva et al. [16] study the problem of *Al*_*2*_*O*_*3*_ nanofluids containing different shaped nanoparticles but they conducted this study experimentally together with theoretical modelling. More exactly they investigated various shapes of *Al*_*2*_*O*_*3*_ nanoparticles in a base fluid mixture of EG and water of equal volumes. By using Hamilton and Crosser model, they noted enough enhancements in the effective thermal conductivities due to particle shapes. Loganathan et al. [17] considered spherical nanoparticles and analyzed radiation effects on an unsteady natural convection flow of nanofluids past an infinite vertical plate. They concluded that spherical silver (*Ag*) nanofluids velocity is less than copper (*Cu*), titanium dioxide (*TiO*_*2*_) and *Al*_*2*_*O*_*3*_ spherical nanofluids due to greater viscosity. Recently, Asma et al. [18] obtained exact solutions for free convection flow of nanofluids with ramped wall temperature by taking five different types of spherical shaped nanoparticles.

Heat transfer due to convection arises in many physical situations. Convection is of three types i.e. free convection, forced convection and mixed convection. The buoyancy induced convection is called free convection whereas forced convection causes due to external pressure gradient or object motion. Mixed convection induces only due to simultaneous occurrence of free and forced convection to transfer heat. The most typical and common situations where mixed convection is almost always realized is the flow in the channel due to the process on heating or cooling the channel walls. In such a flow situation, the buoyancy force causes free convection whereas the external pressure gradient or the non homogeneous boundary conditions on the velocity results forced convection. Sebdani et al. [19] studied heat transfer of *Al*_*2*_*O*_*3*_ water nanofluid in mixed convection flow inside a square cavity. Fan et al. [20] investigated mixed convection heat transfer in a horizontal channel filled with nanofluids. Tiwari and Das [21] and Sheikhzadeh et al. [22] analyzed laminar mixed convection flow of a nanofluid in two-sided lid-driven enclosures. Further, magnetic field in nanofluids has its numerous applications such as in the polymer industry and metallurgy where hydromagnetic techniques are being used. Nadeem and Saleem [23] examined the unsteady flow of a rotating MHD nanofluid in a rotating cone in the presence of magnetic field. Al-Salem et al. [24] investigated MHD mixed convection flow in a linearly heated cavity. The effects of variable viscosity and variable thermal conductivity on the MHD flow and heat transfer over a non-linear stretching sheet was investigated by Prasad et al. [25]. The problem of Darcy Forchheimer mixed convection heat and mass transfer in fluid-saturated porous media in the presence of thermophoresis was presented by Rami et al. [26]. Effect of radiation and magnetic field on the mixed convection stagnation-point flow over a vertical stretching sheet in a porous medium bounded by a stretching vertical plate was presented by Hayat et al. [27]. Few other studies on mixed convection and nanofluids are given in [28–38].

Based on above literature, the present investigation is concerned with the radiative heat transfer in mixed convection MHD flow of a different shapes of *Al*_*2*_*O*_*3*_ in EG-based nanofluid in a channel filled with saturated porous medium. The focus in this work is the effect of different parameters on cylinder shape nanofluids. The fluid is assumed to be electrically conducting and the no slip condition is considered at the boundary of the channel. Three different flow situations are discussed. In the first case, both of the bounding walls of the channel are at rest. Fluid motion is originated due to buoyancy force together with external pressure gradient of oscillatory form applied in the flow direction. In the second case, the upper wall of the channel is set into oscillatory motion whereas the third case extends this idea when both of the channel walls are given oscillatory motions. Analytical solutions are obtained for velocity and temperature profile. The results for skin friction and Nusselt number are computed. Graphical results for velocity field and temperature distributions are displayed for various parameters of interest and discussed in details.

## Formulation and Solution of the Problem

Consider oscillatory flow of an incompressible nanofluids in a channel filled with a saturated porous medium. The fluid is assumed electrically conducting under the influence of a uniform magnetic field of strength B_0_ applied in a transverse direction to the flow. The magnetic Reynolds number is also assumed small enough so that the effect of induced magnetic field can be neglected. The assumptions that the external electric field is considered zero and that the electric field due to polarization is negligible. The no-slip condition at the boundary walls is considered and there is radiation effect in the energy equation. The *x*– axis is taken along the flow and *y*– axis is taken normal to the flow direction. The mixed convection is caused due to buoyancy force together with external pressure gradient applied along the *x*– direction. Under the usual assumption of Boussinesq approximation, the governing equations of momentum and energy as follows:

1 $$ {\rho}_{nf}\frac{\partial u}{\partial t}=-\frac{\partial p}{\partial x}+{\mu}_{nf}\frac{\partial^2u}{\partial {y}^2}-\left(\sigma {B}_0^2+\frac{\mu_{nf}}{k_1}\right)\;u+{\left(\rho \beta \right)}_{nf}g\left(T-{T}_0\right), $$

2 $$ {\left(\rho {c}_p\right)}_{nf}\frac{\partial T}{\partial t}={k}_{nf}\frac{\partial^2T}{\partial {y}^2}-\frac{\partial q}{\partial y}, $$

where *u* = *u*(*y*, *t*) denotes the fluid velocity in the *x*– direction, *T* = *T*(*y*, *t*) is the temperature, *ρ*_*nf*_ is density of nanofluids, *μ*_*nf*_ is the dynamic viscosity of nanofluid, *σ* is the electrical conductivity of the base fluid, *k*_1_ > 0 is the permeability of the porous medium (*ρβ*)_*nf*_, thermal expansion coefficient of nanofluids *g*, is the acceleration due to gravity (*ρc*_*p*_)_*nf*_, is the heat capacitance of nanofluids, *k*_*nf*_ is the thermal conductivity of nanofluid, *q* is the radiative heat flux in *x*– direction. The first term on the right denotes the externa l pressure gradient.

In this study, Hamilton and Crosser model [28], for thermal conductivity and dynamic viscosity is used, being valid for both spherical and non spherical shapes nanoparticles. According to this model:

3 $$ {\mu}_{nf}=\kern1em {\mu}_f\left(1+a\phi +b{\phi}^2\right), $$

4 $$ \frac{k_{nf}}{k_f}=\frac{k_s+\left(n-1\right){k}_f+\left(n-1\right)\left({k}_s-{k}_f\right)\phi }{k_s+\left(n-1\right){k}_f-\left({k}_s-{k}_f\right)\phi }, $$

In equations (1) and (2), the density *ρ*_*nf*_, thermal expansion coefficient (*ρβ*)_*nf*_, heat capacitance (*ρc*_*p*_)_*nf*_ and thermal conductivity of nanofluids are derived by using the relations given by [17, 18] as follows:

5 $$ \begin{array}{c}{\rho}_{nf}=\left(1-\phi \right){\rho}_f+\phi {\rho}_s,\kern0.48em {\left(\rho \beta \right)}_{nf}=\left(1-\phi \right){\left(\rho \beta \right)}_f+\phi {\left(\rho \beta \right)}_s\\ {}{\left(\rho {c}_p\right)}_{nf}=\left(1-\phi \right){\left(\rho {c}_p\right)}_f+\phi {\left(\rho {c}_p\right)}_s,\kern0.36em \end{array} $$

where *ϕ* is the nanoparticles volume fraction, *ρ*_*f*_ and *ρ*_*s*_ are the densities of the base fluid and solid nanoparticles, *β*_*s*_ and *β*_*f*_ are the volumetric coefficients of thermal expansions of solid nanoparticles and base fluids (*c*_*p*_)_*s*_, and (*c*_*p*_)_*f*_ are the specific heat capacities of solid nanoparticles and base fluids at constant pressure *a*, and *b* are constants and depend on the particle shape as given in Table 1 [16].

**Table 1:** Constants *a* and *b* empirical shape factors

Model	Platelet	Blade	Cylinder	Brick
*a*	37.1	14.6	13.5	1.9
*b*	612.6	123.3	904.4	471.4

The *n* appearing in Eq. (4) is the empirical shape factor given by *n* = 3/*Ψ*, where *Ψ* is the sphericity defined as the ratio between the surface area of the sphere and the surface area of the real particle with equal volumes. The values of *Ψ* for different shape particles are given in Table 2 [16].

**Table 2:** Sphericity *Ψ* for different shapes nanoparticles

Model	Platelet	Blade	Cylinder	Brick
*Ψ*	0.52	0.36	0.62	0.81

In addition to above, some physical properties of base fluid and nanoparticles are given in Table 3 as mentioned by [17] and [18].

**Table 3:** Thermophysical properties of water and nanoparticles

Model	*ρ*(*kgm* ^− 3^)	*c*_*P*_(*kg* ^− 1^ *K* ^− 1^)	*k*(*Wm* ^− 1^ *K* ^− 1^)	*β* × 10^− 5^(*K* ^− 1^)
*H*_*2*_ *O*	997.1	4179	0.613	21
*C*_*2*_ *H*_*6*_ *O*_*2*_	1.115	0.58	0.1490	6.5
*Cu*	8933	385	401	1.67
*TiO*_*2*_	4250	686.2	8.9528	0.9
*Ag*	10500	235	429	1.89
*Al*_*2*_ *O*_*3*_	3970	765	40	0.85
*Fe*_*3*_ *O*_*4*_	5180	670	9.7	0.5

Following Makinde and Mhone [31], both plates temperature *T*_0_ and *T*_*w*_ are assumed high enough and produces the radiative heat transfer. Thus, the radiative heat flux is given by

6 $$ \frac{\partial q}{\partial y}=-4{\alpha}^2\left(T-{T}_0\right), $$

where *α* is the radiation absorption coefficient.

Substituting Eq. (6) into Eq. (2), gives

7 $$ {\left(\rho {c}_p\right)}_{nf}\frac{\partial T}{\partial t}={k}_{nf}\frac{\partial^2T}{\partial {y}^2}+4{\alpha}^2\left(T-{T}_0\right), $$

where *α* is the mean radiation absorption coefficient.

Introducing the following dimensionless variables

8 $$ \begin{array}{l}{x}^{\ast }=\frac{x}{d},\;{y}^{\ast }=\frac{y}{d},\kern0.5em {u}^{\ast }=\frac{u}{U_0},\kern0.62em {t}^{\ast }=\frac{t{U}_0}{d},\kern0.62em {p}^{\ast }=\frac{d}{\mu {U}_0}p,\;\\ {}\kern0.5em {T}^{\ast }=\frac{T-{T}_0}{T_w-{T}_0},\kern0.62em {\omega}^{\ast }=\frac{d\omega }{U_0},\kern0.24em \frac{\partial {p}^{\ast }}{\partial {x}^{\ast }}=\lambda exp\left(i{\omega}^{*}{t}^{*}\right)\end{array} $$

into Eqs. (1) and (7), give (*symbol is dropped for convenience)

9 $$ \begin{array}{c}\left[\left(1-\phi \right)+\phi \frac{\rho_s}{\rho_f}\right]\;Re\frac{\partial u}{\partial t}=\lambda \varepsilon exp\left(i\omega t\right)+\left(1+a\phi +b{\phi}^2\right)\frac{\partial^2u}{\partial {y}^2}-{M}^2u\\ {}-\frac{\left(1+a\phi +b{\phi}^2\right)u}{K}+\left[\left(1-\phi \right)+\phi \frac{{\left(\rho \beta \right)}_s}{{\left(\rho \beta \right)}_f}\right]GrT,\;\end{array} $$

10 $$ Pe\frac{\phi_4}{\lambda_n}\frac{\partial T}{\partial t}=\frac{\partial^2T}{\partial {y}^2}+\frac{N^2}{\lambda_n}T, $$

where

$$ \begin{array}{c}Re=\frac{U_0d}{v_f},\kern0.62em {M}^2=\frac{\sigma {B}_0^2{d}^2}{\mu_f},\;K=\frac{k_1}{d^2},\kern0.62em Gr=\frac{g{\beta}_f{d}^2\left({T}_w-{T}_0\right)}{\nu_f{U}_0},\\ {}Pe=\frac{U_0d{\left(\rho {c}_p\right)}_f}{k_f},\;{N}^2=\frac{4{d}^2{\alpha}^2}{k_f},\kern0.62em {\lambda}_n=\frac{k_{nf}}{k_f}.\end{array} $$

are the Reynold's number , the magnetic parameter also called Hartmann number, the permeability parameter, the thermal Grashof number, the Peclet number, the radiation parameter, and

$$ {\phi}_1=\left(1-\phi \right)+\phi \frac{\rho_s}{\rho_f},\kern0.36em {\phi}_2=\left(1+a\phi +b{\phi}^2\right),\kern0.24em {\phi}_3=\left(1-\phi \right){\rho}_f+\phi \frac{{\left(\rho \beta \right)}_s}{\beta_f}, $$

$$ {\phi}_4=\left[\left(1-\phi \right)+\phi \frac{{\left(\rho {c}_p\right)}_s}{{\left(\rho {c}_p\right)}_f}\right]. $$

In order to solve Eqs. (9) and (10), we consider the following three cases.

### Case-I: Flow Inside a Channel with Stationary Walls

In the first case, the flow inside a channel of width *d* filled with nanofluids is considered. Both of the walls of the channel are kept stationary at *y* = 0 and *y* = *d*. The upper wall of the channel is assumed maintained at constant temperature *T*_*w*_ and the lower wall has uniform temperature *T*_0_. Thus, the boundary conditions are

11 $$ u\left(0,\;t\right)=0,\kern1.12em u\left(d,\;t\right)=0, $$

12 $$ T\left(0,\;t\right)={T}_0,\kern1.12em T\left(d,\;t\right)={T}_w. $$

In dimensionless form Eqs. (11) and (12) are

13 $$ T\left(0,\;t\right)=0;\kern1.62em T\left(1,\;t\right)=1;\kern1.62em t>0, $$

14 $$ u\left(0,\;t\right)=0;\kern1.62em u\left(1,\;t\right)=0,\kern0.62em t>0. $$

After simplification, Eqs. (9) and (10), take the forms

15 $$ {a}_0\frac{\partial u}{\partial t}=\lambda \varepsilon exp\left(i\omega t\right)+{\phi}_2\frac{\partial^2u}{\partial {y}^2}-{m}_0^2u+{a}_1T, $$

16 $$ {b_0}^2\frac{\partial T}{\partial t}=\frac{\partial^2T}{\partial {y}^2}+{b_1}^2T, $$

where

$$ {a}_0={\phi}_1Re,\kern0.62em {m}_0^2={M}^2+\frac{\phi_2}{K},\kern0.24em {a}_1={\phi}_3Gr,\kern0.24em {b_0}^2=\frac{Pe{\phi}_4}{\lambda_n},\kern0.62em {b_1}^2=\frac{N^2}{\lambda_n}. $$

Now to solve Eqs. (15) and (16) with boundary conditions (13) and (14), the perturbed solutions are taken of the forms:

17 $$ u\left(y,\;t\right)=\left[{u}_0(y)+\varepsilon exp\left(i\omega t\right)\;{u}_1(y)\right], $$

18 $$ T\left(y,\;t\right)=\left[{T}_0(y)+\varepsilon exp\left(i\omega t\right)\;{T}_1(y)\right], $$

for velocity and temperature respectively.

Using Eqs. (17) and (18) into Eqs. (15) and (16), we obtain the following system of ordinary differential equations

19 $$ \frac{d^2{u}_0(y)}{d{y}^2}-{m}_1^2{u}_0(y)=-{a}_2{T}_0(y), $$

20 $$ \frac{d^2{u}_1}{d{y}^2}-{m}_2^2{u}_1(y)=-\lambda, $$

21 $$ \frac{d^2{T}_0(y)}{d{y}^2}+{b_1}^2{T}_0(y)=0, $$

22 $$ \frac{d^2{T}_1(y)}{d{y}^2}+{m}_3^2{T}_1(y)=0, $$

where

$$ {m}_1=\frac{m_0^2}{\phi_2},\;{a}_2=\frac{a_1}{\phi_2},\;{m}_2=\sqrt{\frac{m_0^2+i\omega {a}_0}{\phi_2}},\kern0.62em {m}_3=\sqrt{{b_1}^2-{b_0}^2i\omega }. $$

The associated boundary conditions (13) and (14) are reduce to

23 $$ {u}_0(0)=0;\kern0.62em {u}_0(1)=0, $$

24 $$ {u}_1(0)=0;\kern0.62em {u}_1(1)=0, $$

25 $$ {T}_0(0)=0;\kern0.62em {T}_0(1)=1, $$

26 $$ {T}_1(0)=0;\kern0.62em {T}_1(1)=0. $$

Solutions of Eqs. (21) and (22) under boundary conditions (25) and (26) yield to

27 $$ {T}_0(y)=\frac{sin{b}_1y}{sin{b}_1}, $$

28 $$ {T}_1(y)=0. $$

Eq. (18) using Eqs. (27) and (28), gives

29 $$ T\left(y,\;t\right)=T(y)=\frac{sin{b}_1y}{sin{b}_1}. $$

Eqs. (19) and (20), using Eq. (27) under boundary conditions (23) and (24), give

30 $$ {u}_0(y)={c}_1 sinh\left({m}_1y\right)+{c}_2 cosh{m}_1y+\frac{a_2}{\left({b}_1^2+{m}_1^2\right)}\frac{sin{b}_1y}{sin{b}_1}, $$

31 $$ {u}_1(y)={c}_3 sinh{m}_2y+{c}_4 cosh{m}_2y+\frac{\lambda }{m_2^2{\phi}_2}, $$

with arbitrary constants

32 $$ {c}_1=-\frac{a_2}{sinh{m}_1\left({b}_1^2+{m}_1^2\right)},\;{c}_2=0,\kern0.62em {c}_3=\frac{\lambda }{m_2^2{\phi}_2 sinh{m}_2}\left( cosh{m}_2-1\right),\kern1.12em {c}_4=-\frac{\lambda }{m_2^2{\phi}_2}. $$

Finally, substituting Eqs. (30)-(32), into Eq. (17), we obtained:

33 $$ \begin{array}{c}u\left(y,\;t\right)=-\frac{a_2 sin h{m}_1y}{\left({b}_1^2+{m}_1^2\right) sin h{m}_1}+\frac{a_2 sin{b}_1y}{\left({b}_1^2+{m}_1^2\right) sin{b}_1}\\ {}+\varepsilon \exp \left(i\omega t\right)\;\left[\frac{\lambda \left( cosh{m}_2-1\right) sin h{m}_2y}{m_2^2{\phi}_2 sin h{m}_2}-\frac{\lambda }{m_2^2{\phi}_2}\left( cosh{m}_2y-1\right)\right].\end{array} $$

### Case-2: Flow Inside a Channel with Oscillating Upper Plate

Here the upper wall of the channel (at *y* = *d* ) is set into oscillatory motion while the lower wall (at *y* = 0), is held stationary. The first boundary condition is the same as in Case-1, whereas the second boundary condition in dimensionless form modifies to

34 $$ u\left(1,\;t\right)=H(t)\varepsilon \exp \left(i\omega t\right);\kern0.62em t>0, $$

where *H*(*t*) is the Heaviside step function.

By using the same procedure as in Case-1, and the solution is obtained as

35 $$ \begin{array}{c}u\left(y,\;t\right)=-\frac{a_2 sin h{m}_1y}{\left({b}_1^2+{m}_1^2\right) sin h{m}_1}+\frac{a_2 sin{b}_1y}{\left({b}_1^2+{m}_1^2\right) sin{b}_1}\\ {}+\varepsilon exp\left(i\omega t\right)\;\left[\begin{array}{c}\hfill {\scriptscriptstyle \frac{sinh{m}_2y}{sinh{m}_2}}\left\{H(t)+{\scriptscriptstyle \frac{\lambda }{m_2^2{\phi}_2}}\left( cosh{m}_2-1\right)\right\}-{\scriptscriptstyle \frac{\lambda }{m_2^2{\phi}_2}} cosh{m}_2y+{\scriptscriptstyle \frac{\lambda }{m_2^2{\phi}_2}}\hfill \\ {}\hfill \hfill \end{array}\right].\end{array} $$

### Case-3: Flow Inside a Channel with Oscillating Upper and Lower Plates

In this case both of the channel walls are set into oscillatory motions. The dimensionless form of the boundary conditions is

36 $$ u\left(0,\;t\right)=u\left(1,\;t\right)=H(t)\varepsilon exp\left(i\omega t\right);\kern0.62em t>0. $$

The resulting expression for velocity is obtained as:

37 $$ \begin{array}{c}u\left(y,\;t\right)=-\frac{a_2 sin h{m}_1y}{sinh{m}_1\left({b}_1^2+{m}_1^2\right)}+\frac{a_2 sin{b}_1y}{\left({b}_1^2+{m}_1^2\right) sin{b}_1}\\ {}+\varepsilon exp\left(i\omega t\right)\;\left[\begin{array}{c}\hfill {\scriptscriptstyle \frac{\left( cosh{m}_2-1\right) sin h{m}_2y}{sinh{m}_2}}\left({\scriptscriptstyle \frac{\lambda }{m_2^2{\phi}_2}}-H(t)\right)+\left(H(t)-{\scriptscriptstyle \frac{\lambda }{m_2^2{\phi}_2}}\right) cosh{m}_2y+{\scriptscriptstyle \frac{\lambda }{m_2^2{\phi}_2}}\hfill \\ {}\hfill \hfill \end{array}\right].\end{array} $$

### Nusselt Number and Skin-friction

The dimensionless expressions for Nusselt number and skin-frictions are evaluated from Eqs. (29), (33), (35) and (37) are as follows:

38 $$ Nu=-\frac{b_1}{sin{b}_1}, $$

39 $$ {\tau}_1={\tau}_1(t)=\frac{a_2{m}_1}{\left({b}_1^2+{m}_1^2\right) sin h{m}_1}-\frac{b_1{a}_2}{\left({b}_1^2+{m}_1^2\right) sin{b}_1}+\varepsilon exp\left(i\omega t\right)\;\left[\frac{\lambda \left(1- cosh{m}_2\right)}{m_2{\phi}_2 sinh{m}_2}\right], $$

40 $$ \begin{array}{c}{\tau}_2={\tau}_2(t)=\frac{a_2{m}_1}{\left({b}_1^2+{m}_1^2\right) sin h{m}_1}-\frac{b_1{a}_2}{\left({b}_1^2+{m}_1^2\right) sin{b}_1}\\ {}+\varepsilon \exp \left(i\omega t\right)\;\left[\frac{m_2}{sinh{m}_2}\left\{H(t)+\frac{\lambda }{m_2^2{\phi}_2}\Big( cosh{m}_2-1\right\}\right],\;\end{array} $$

41 $$ \begin{array}{c}{\tau}_3={\tau}_3(t)=\frac{a_2{m}_1}{sinh{m}_1\left({b}_1^2+{m}_1^2\right)}-\frac{a_2{b}_1}{\left({b}_1^2+{m}_1^2\right) sin{b}_1}\\ {}+\varepsilon exp\left(i\omega t\right)\;\left[\frac{m_2\left( cosh{m}_2-1\right)}{sinh{m}_2}\left(H(t)-\frac{\lambda }{m_2^2{\phi}_2}\right)\right].\end{array} $$

## Graphical Results and Discussion

**Fig. 1:**
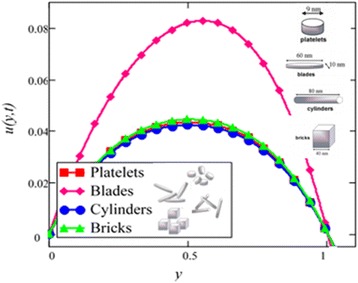
Velocity profile of different shapes of *Al*_2_ *O*_3_ nanoparticles in EG-based nanofluid when *Gr* = 0.1, *N* = 0.1, *φ* = 0.04, *λ* = 1, *K* = 1, *M* = 1, *t* = 5, *ω* = 0.2.

**Fig. 2:**
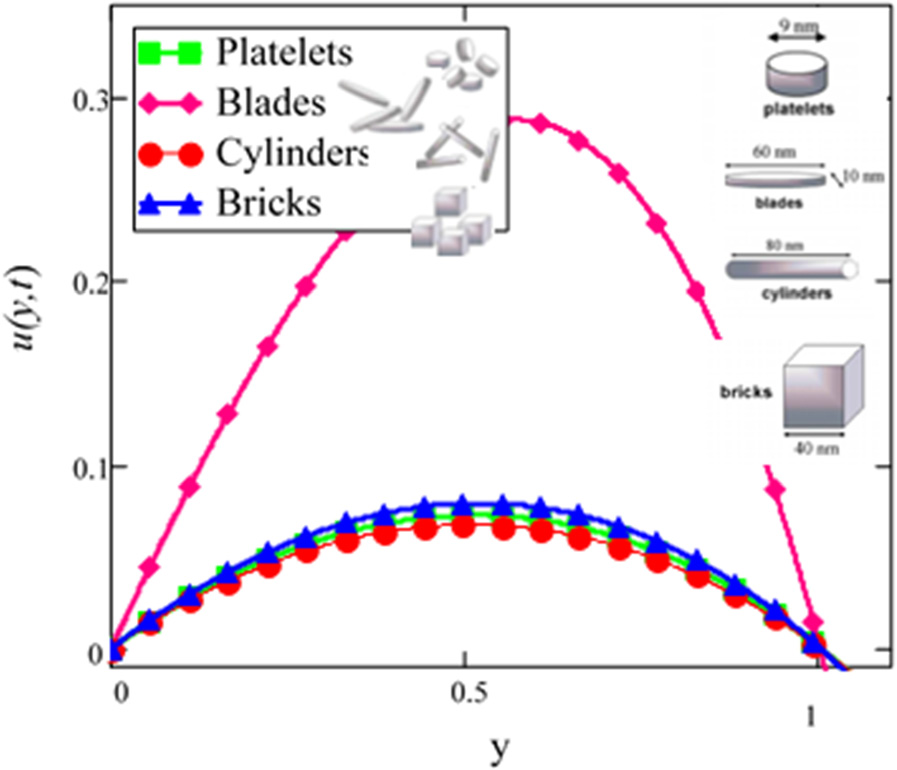
Velocity profile of different shapes of *Al*_2_ *O*_3_ nanoparticles in water-based nanofluid when *Gr* = 0.1, *N* = 0.1, *φ* = 0.04, *λ* = 1, *M* = 1, *K* = 1, *t* = 5, *ω* = 0.2.

**Fig. 3:**
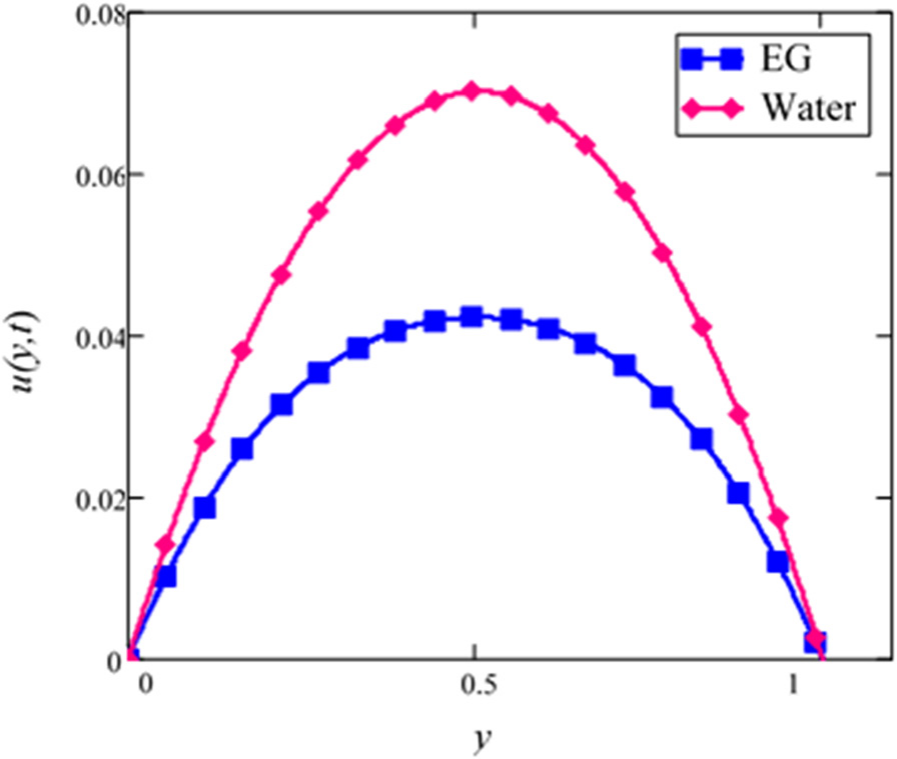
Velocity comparison graph of EG and water-based nanofluids when *Gr* = 0.1, *N* = 0.1, *φ* = 0.04, *λ* = 1, *M* = 1, *K* = 1, *t* = 5, *ω* = 0.2.

**Fig. 4:**
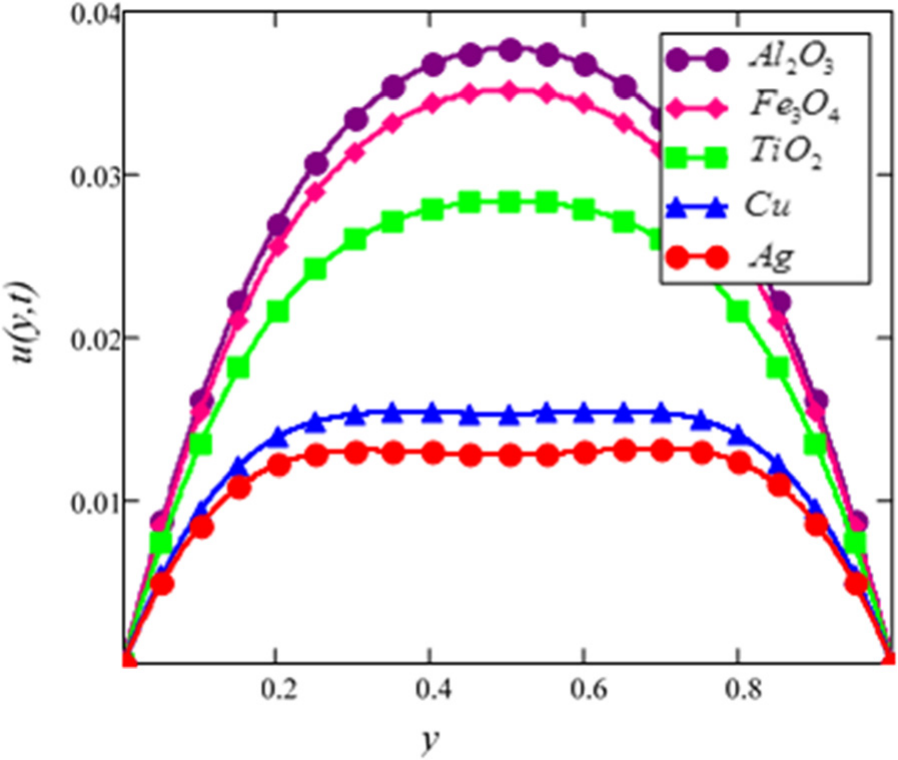
Velocity profile of different nanoparticles in EG-based nanofluid when *Gr* = 0.1, *N* = 0.1, *φ* = 0.04, *λ* = 1, *M* = 1, *t* = 5, *K* = 2, *ω* = 0.2.

**Fig. 5:**
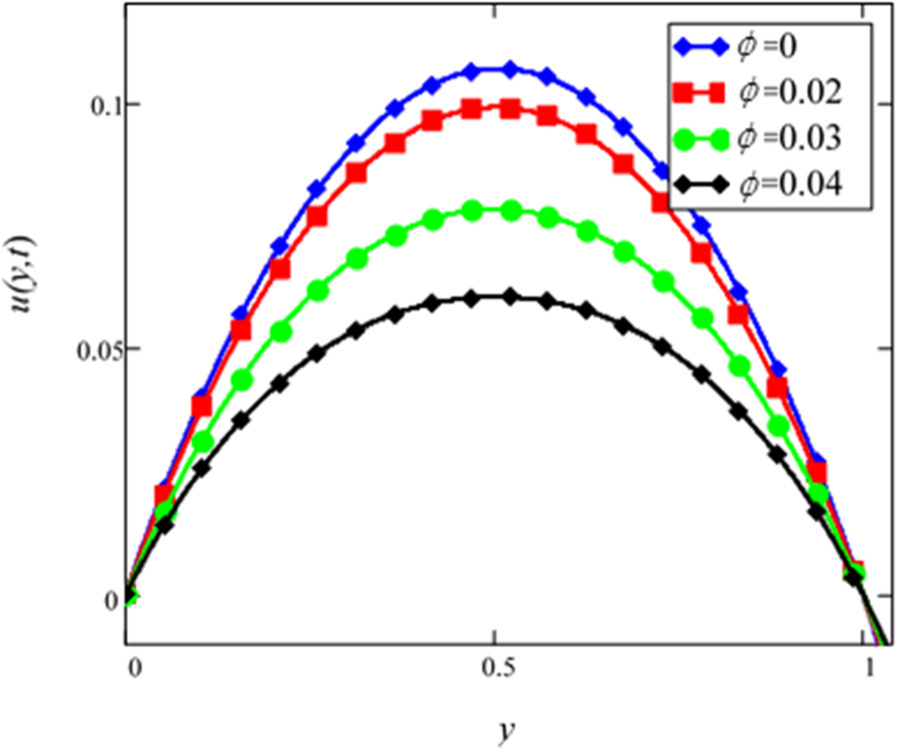
Velocity profile of different ϕ of *Al*_2_ *O*_3_ in EG-based nanofluid when *Gr* = 0.1, *N* = 0.1, *λ* = 1, *M* = 1, *K* = 1, *t* = 5, *ω* = 0.2.

**Fig. 6:**
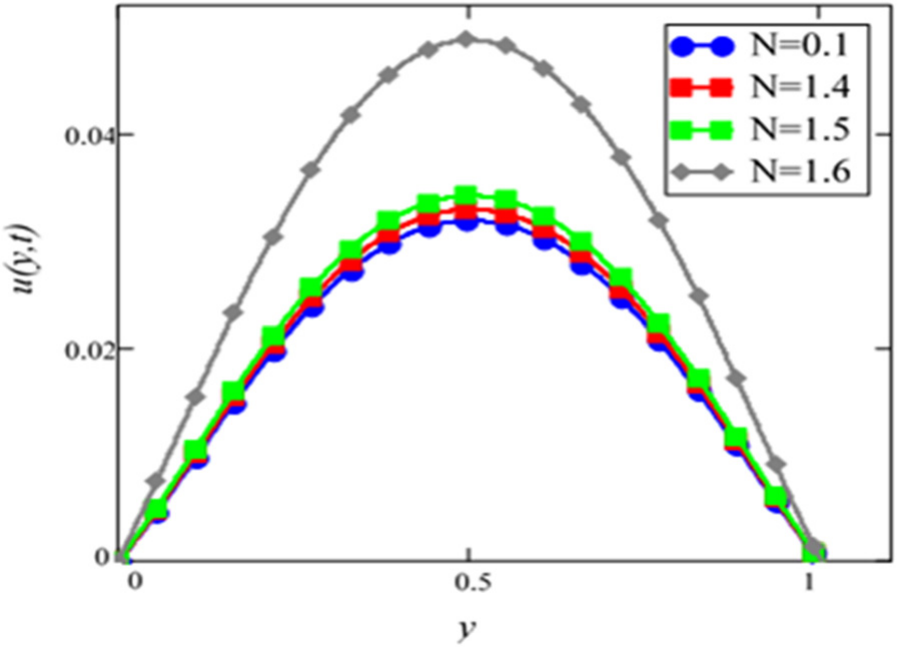
Velocity profile for different values of *N* in EG-based nanofluid when *Gr* = 0.1, *φ* = 0.04, *λ* = 1, *M* = 1, *K* = 1, *t* = 10, *ω* = 0.2.

**Fig. 7:**
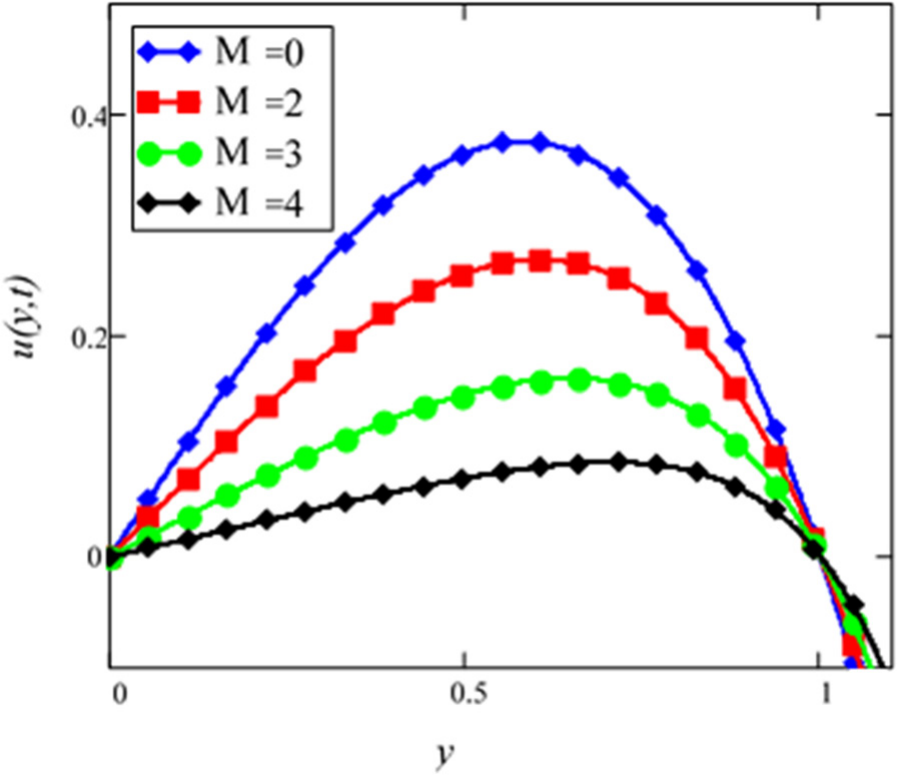
Velocity profile for different values of *M* in EG-based nanofluid when *Gr* = 0.1, *N* = 0.1, *φ* = 0.04, *λ* = 1, *K* = 1, *t* = 10, *ω* = 0.2.

**Fig. 8:**
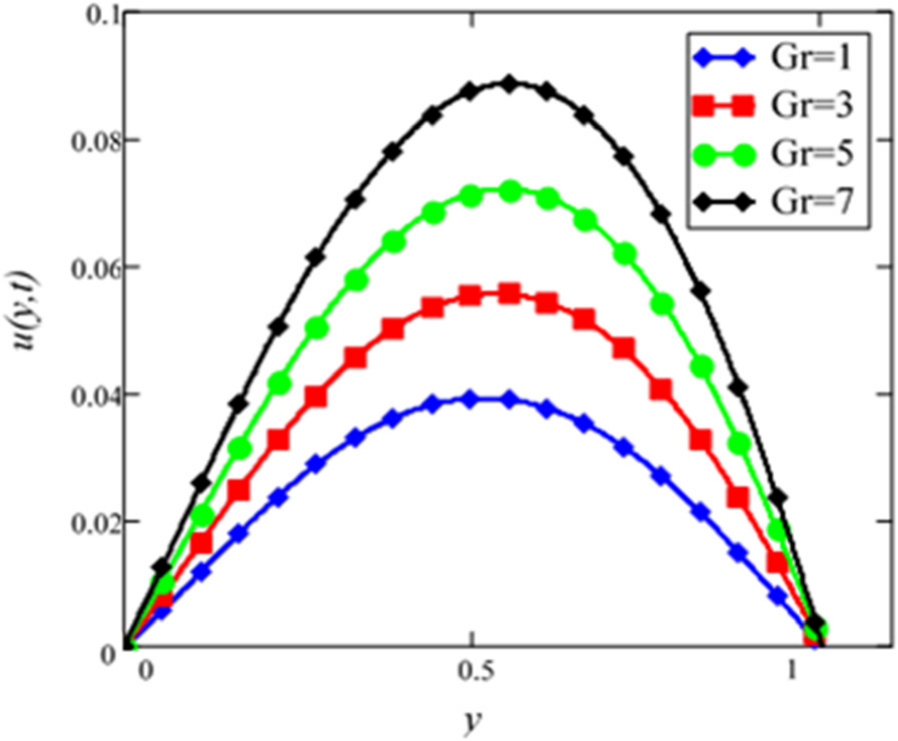
Velocity profile for different values of *Gr* in EG-based nanofluid when *N* = 0.1, *φ* = 0.04, *λ* = 1, *M* = 1, *K* = 1, *t* = 10, *ω* = 0.2.

**Fig. 9:**
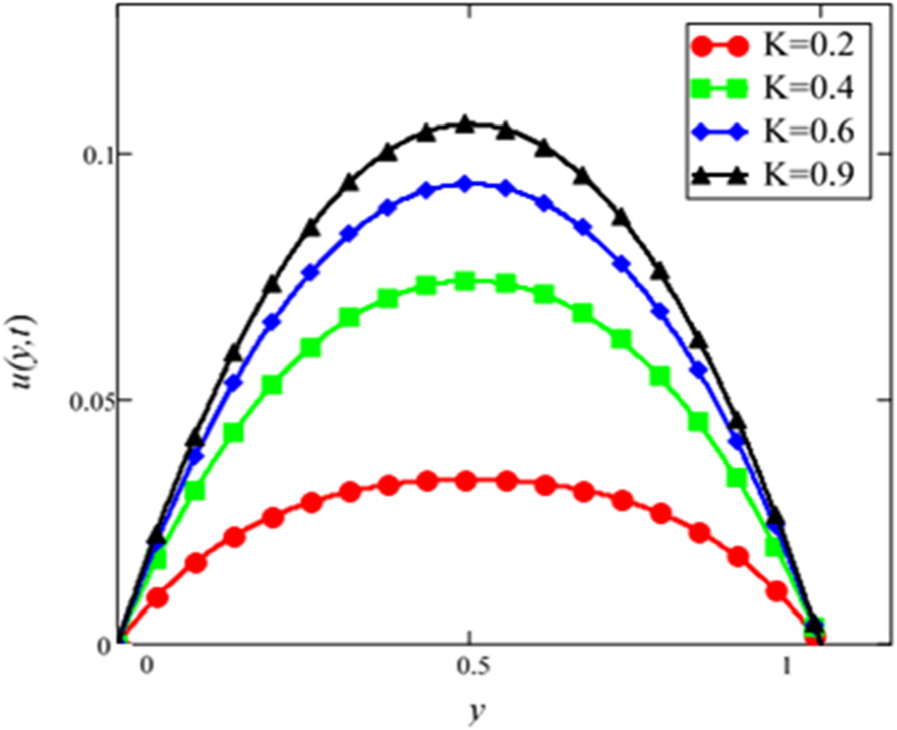
Velocity profile of different value of *K* in EG-based nanofluid when *Gr* = 0.1, *N* = 0.1, *φ* = 0.04, *λ* = 0.01, *M* = 1, *t* = 5, *ω* = 0.2.

**Fig. 10:**
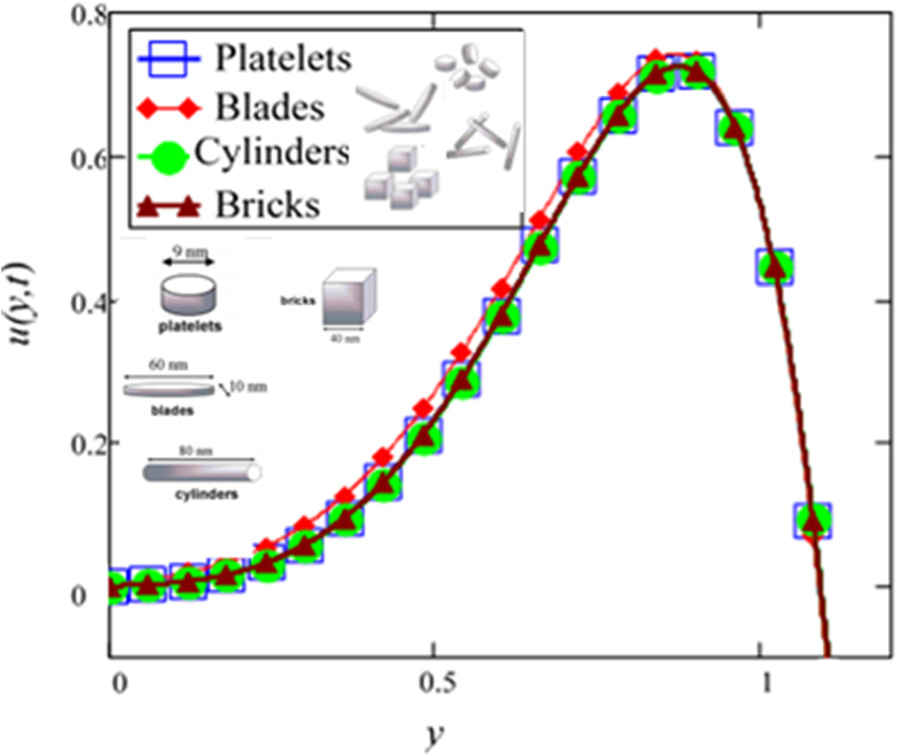
Velocity profile of different shapes of *Al*_2_ *O*_3_ nanoparticles in EG-based nanofluid when *Gr* = 0.1, *N* = 0.1, *φ* = 0.04, *λ* = 0.01, *M* = 1, *K* = 1, *t* = 5, *ω* = 0.2.

**Fig. 11:**
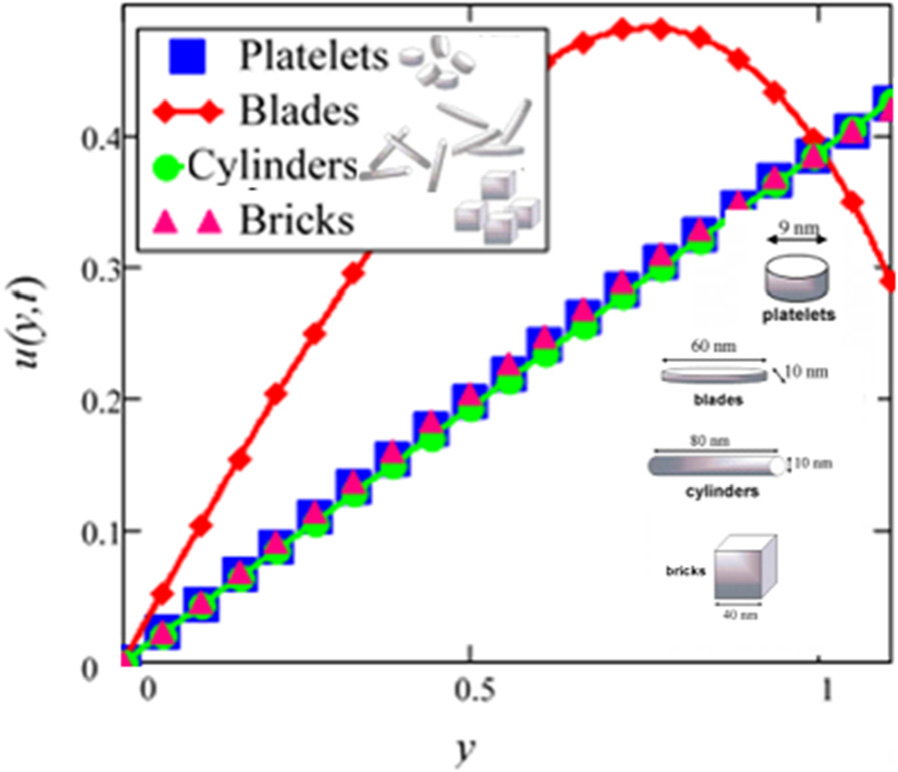
Velocity profile of different shapes of *Al*_2_ *O*_3_ nanoparticles in water-based nanofluid when *Gr* = 0.1, *N* = 0.1, *φ* = 0.04, *λ* = 0.01, *M* = 1, *t* = 5, *K* = 1, *ω* = 0.2.

**Fig. 12:**
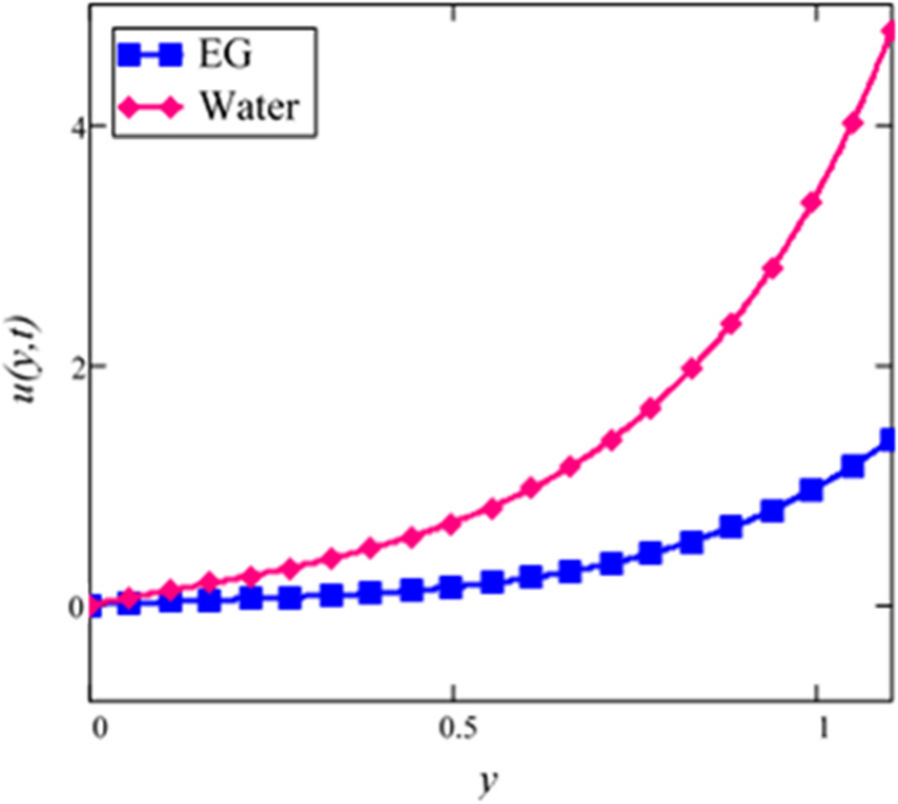
Velocity comparison graph of EG and water-based nanofluids when *Gr* = 0.1, *N* = 0.1, *φ* = 0.04, *λ* = 0.01, *M* = 1, *K* = 1, *t* = 5, *ω* = 0.2.

**Fig. 13:**
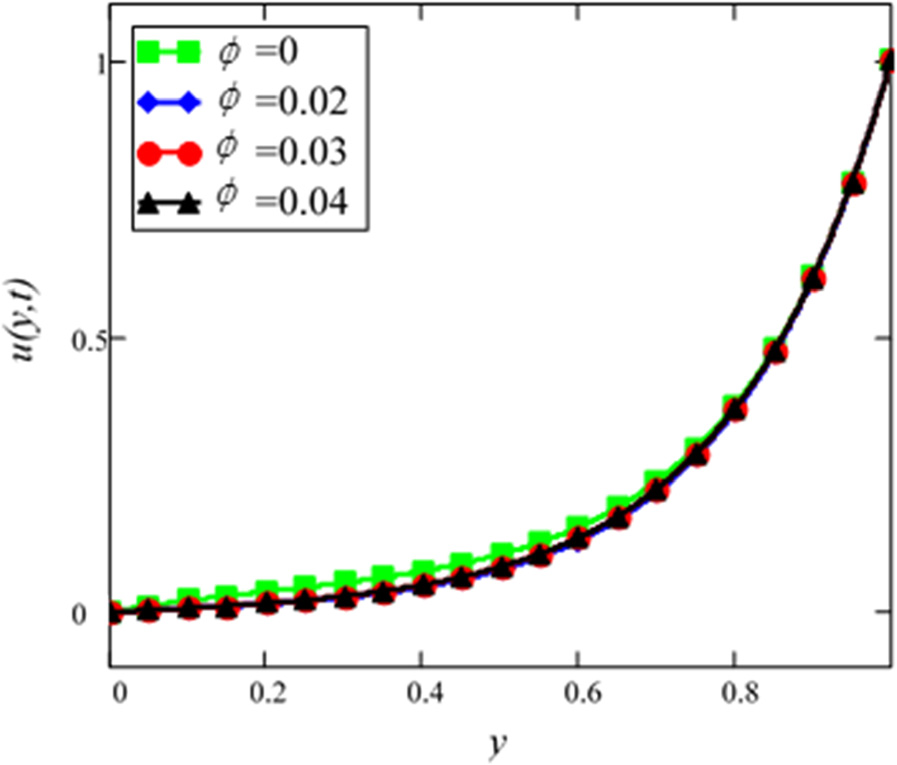
Velocity profile of different ϕ of *Al*_2_ *O*_3_in EG-based nanofluid *Gr* = 0.1, *N* = 0.1, *φ* = 0.04, *λ* = 0.01, *M* = 1, *K* = 1, *t* = 5, *ω* = 0.2.

**Fig. 14:**
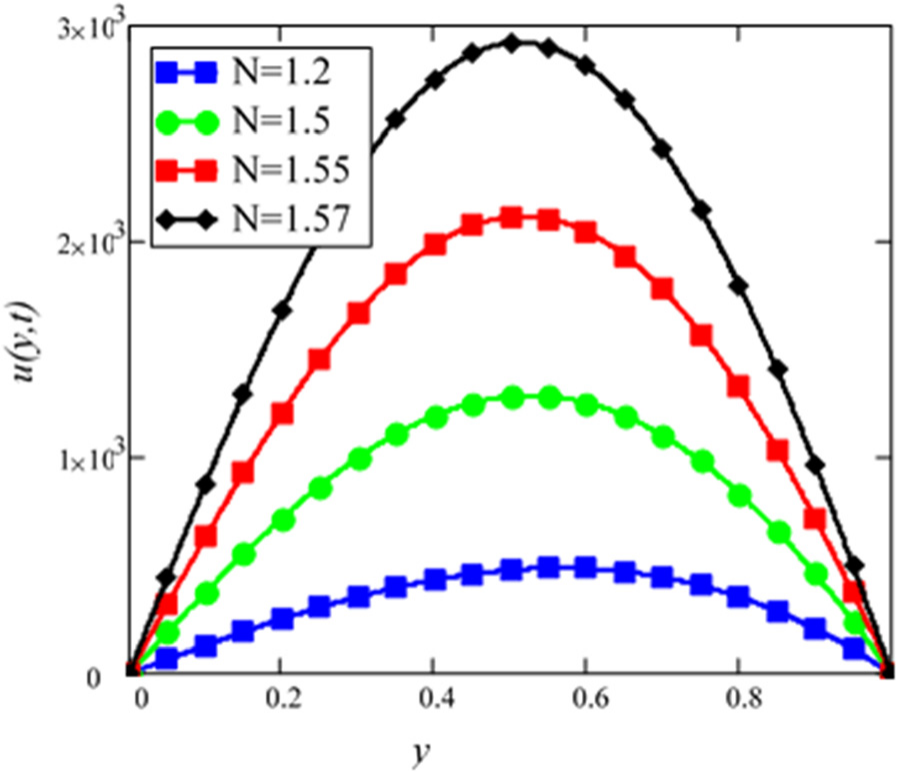
Velocity profile for different values of *N* in EG-based nanofluid when *Gr* = 1, *φ* = 0.04, *λ* = 0.01, *M* = 1, *K* = 0.2, *t* = 10, *ω* = 0.2.

**Fig. 15:**
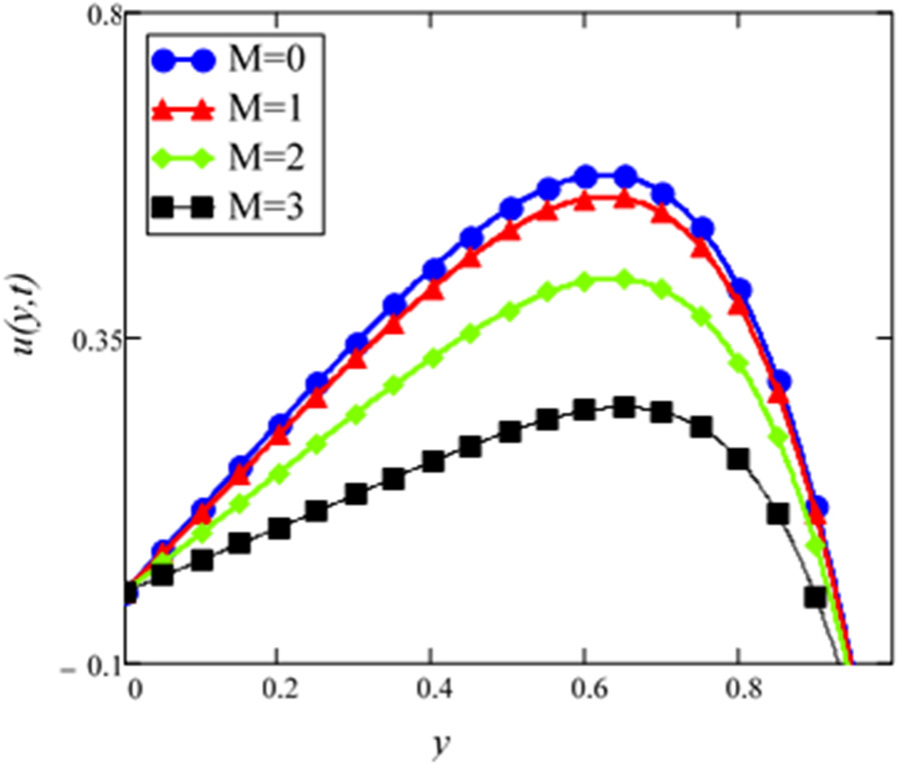
Velocity profile for different values of *M* in EG-based nanofluid when *Gr* = 1, *N* = 0.1, *φ* = 0.04, *λ* = 0.001, *K* = 1, *t* = 10, *ω* = 0.2.

**Fig. 16:**
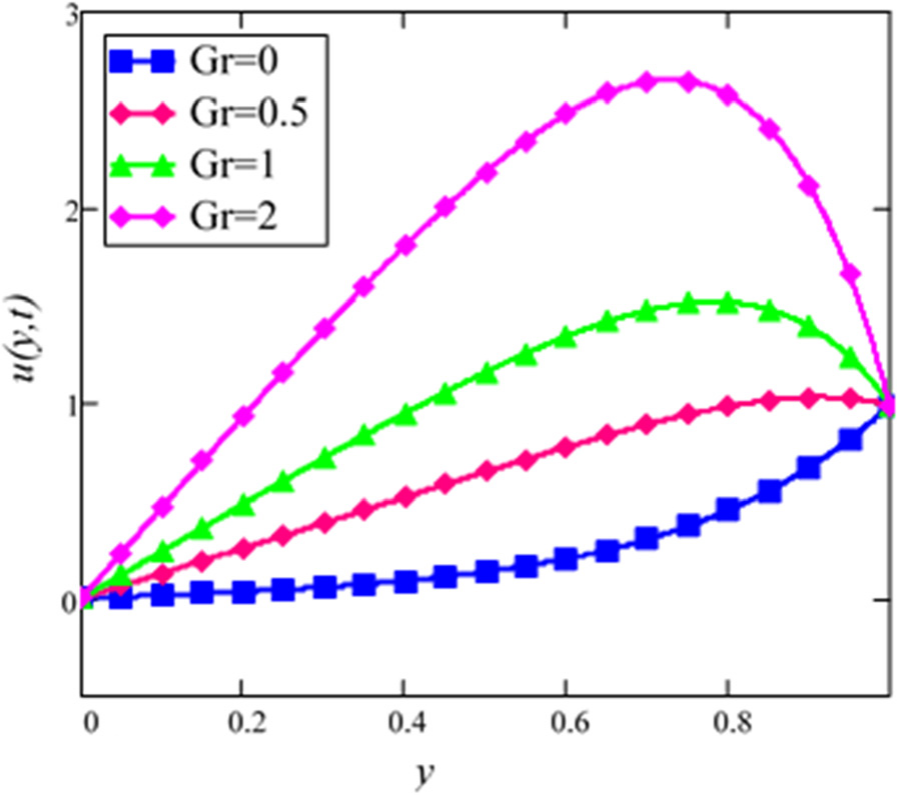
Velocity profile for different values of *Gr* in EG-based nanofluid when *N* = 0.1, *φ* = 0.04, *λ* = 0.01, *M* = 1, *K* = 0.2, *t* = 10, *ω* = 0.2.

**Fig. 17:**
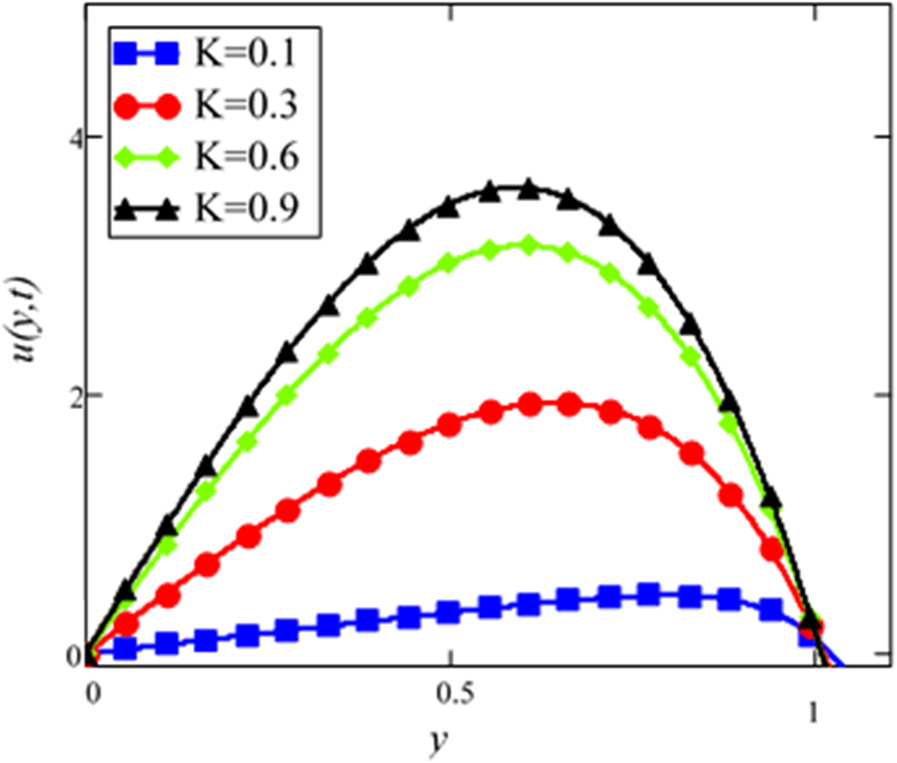
Velocity profile for different values of *K* in EG-based nanofluid when *N* = 0.1, *φ* = 0.04, *λ* = 0.01, *M* = 1, *t* = 10, *ω* = 0.2.

**Fig. 18:**
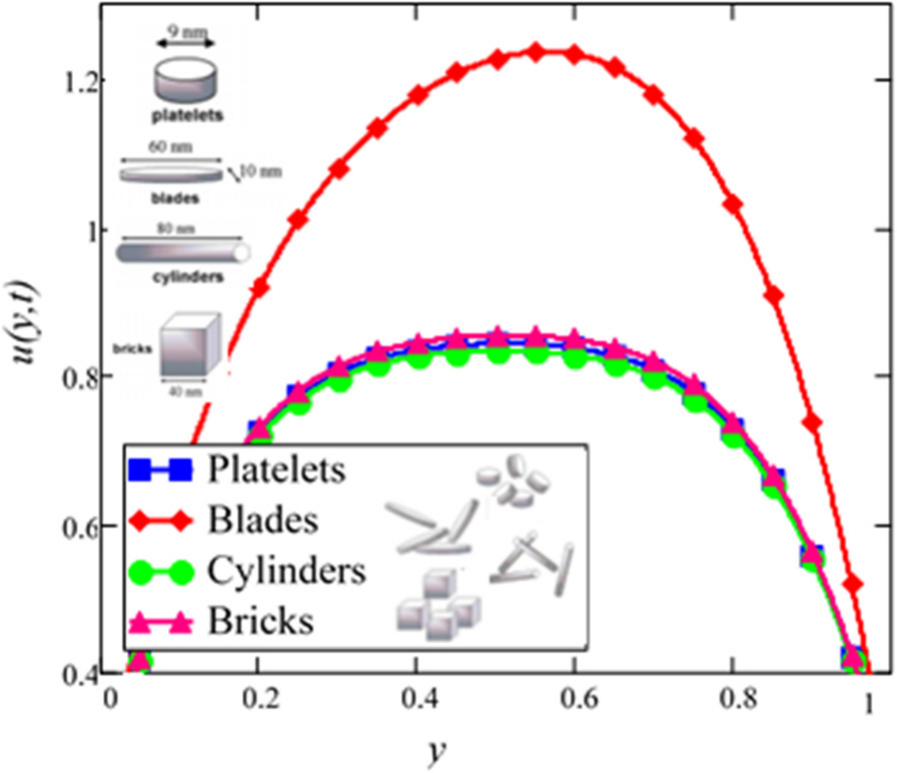
Velocity profile of different shapes of *Al*_2_ *O*_3_ nanoparticles in EG-based nanofluid when *Gr* = 1, *N* = 0.1, *φ* = 0.04, *λ* = 0.01, *M* = 1, *K* = 1, *t* = 10, *ω* = 0.2.

**Fig. 19:**
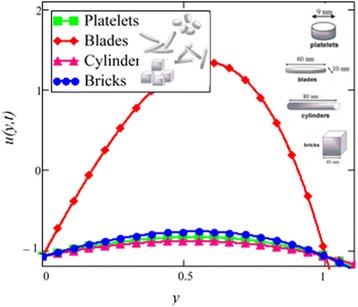
Velocity profile of different shapes of *Al*_2_ *O*_3_ nanoparticles in water-based nanofluid when *Gr* = 1, *N* = 0.1, *φ* = 0.04, *λ* = 0.01, *M* = 1, *K* = 1, *t* = 10, *ω* = 0.2.

**Fig. 20:**
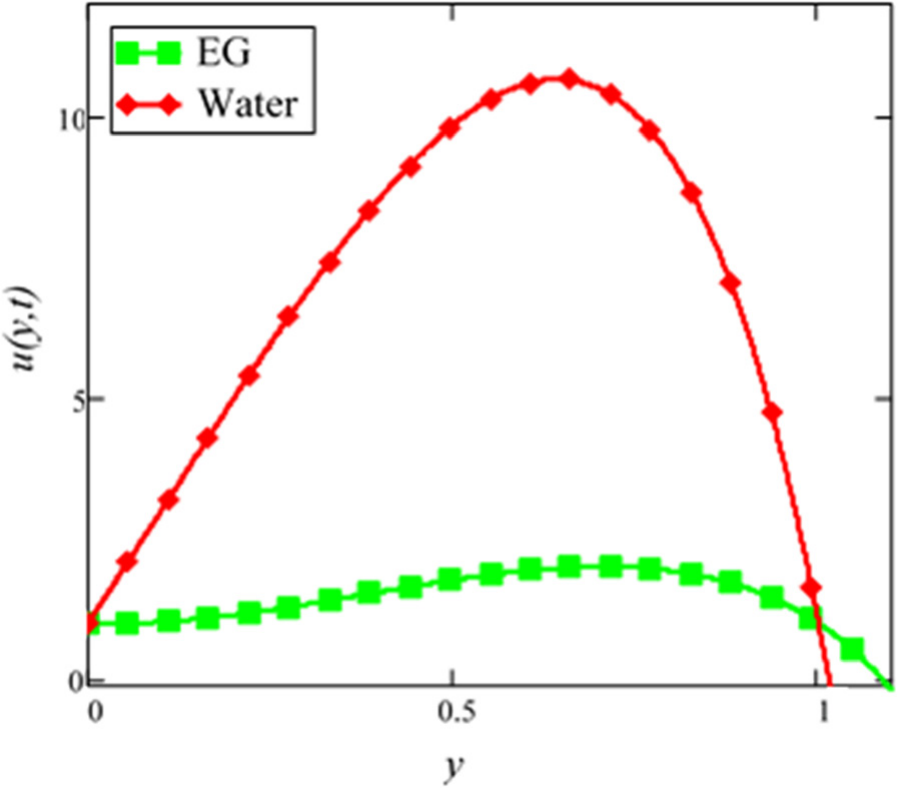
Velocity comparison graph of EG and water-based nanofluids when *Gr* = 1, *N* = 0.1, *φ* = 0.04, *λ* = 0.01, *M* = 1, *K* = 1, *t* = 10, *ω* = 0.2.

**Fig. 21:**
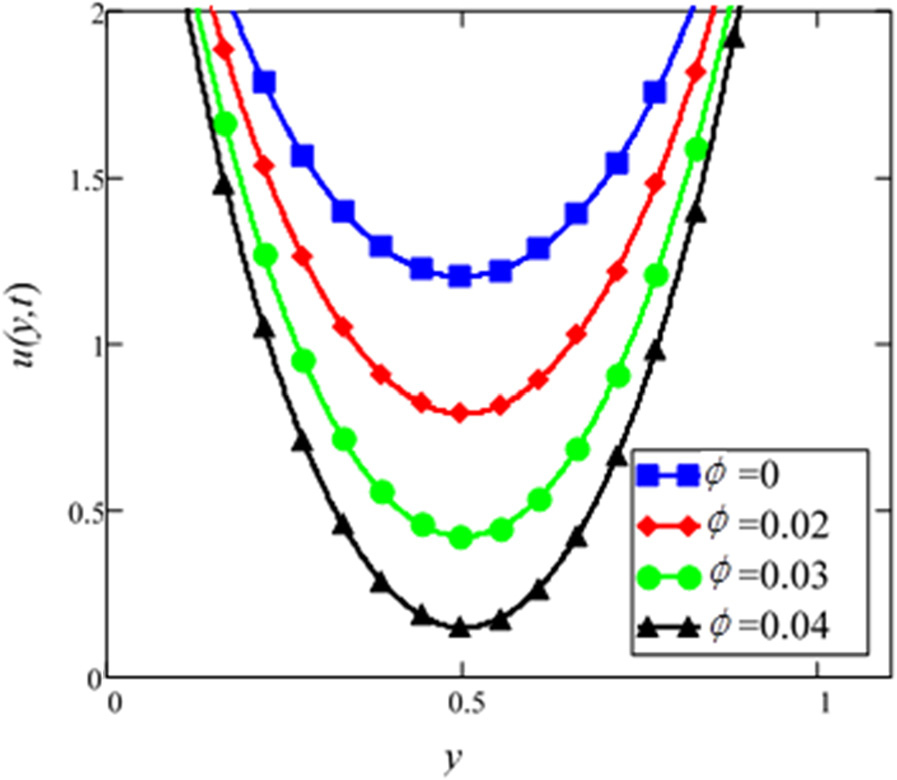
Velocity profile for different values of *ϕ* in EG-based nanofluid when *Gr* = 1, *N* = 0.1, *λ* = 0.01, *M* = 1, *K* = 1, *t* = 10, *ω* = 0.2.

**Fig. 22:**
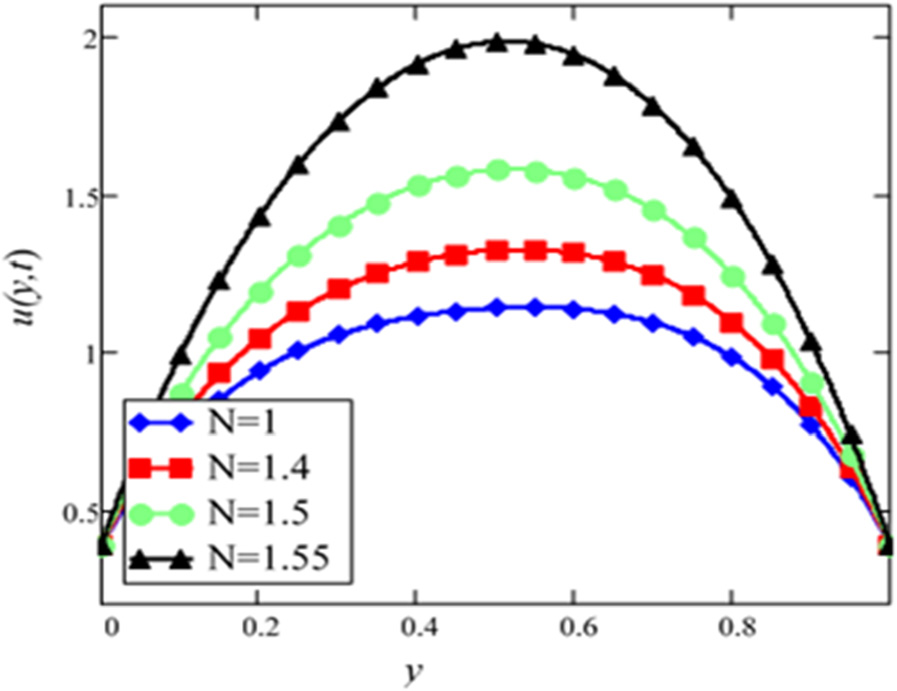
Velocity profile for different values of *N* in EG-based nanofluid when *Gr* = 1, *φ* = 0.04, *λ* = 0.01, *M* = 1, *K* = 1, *t* = 10, *ω* = 0.2.

**Fig. 23:**
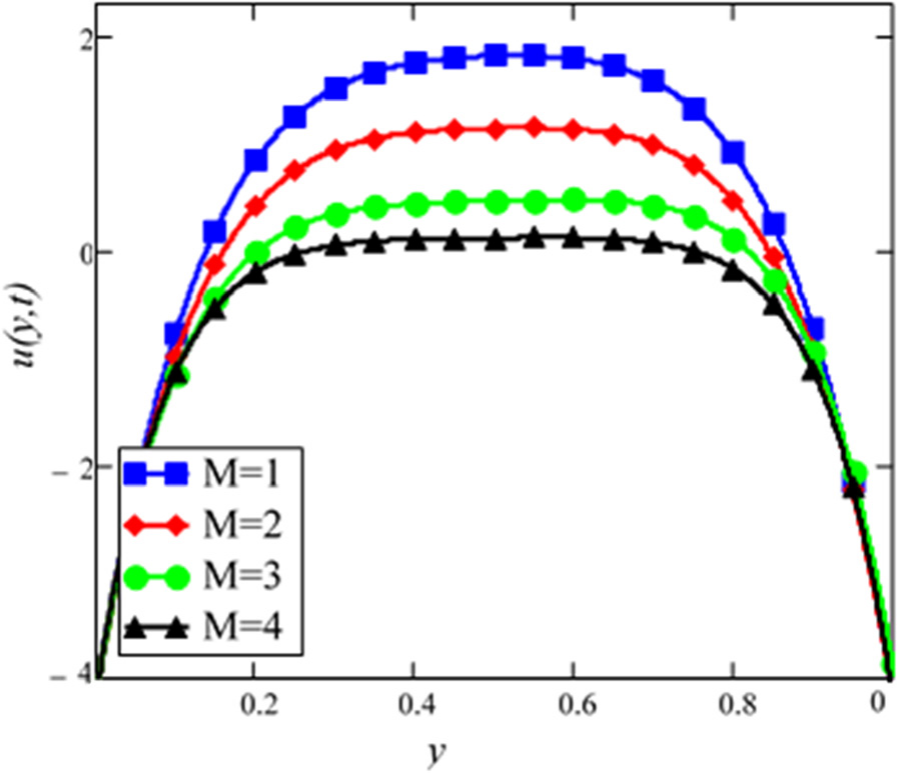
Velocity profile for different values of *M* in EG-based nanofluid when *Gr* = 1, *N* = 0.1, *φ* = 0.04, *λ* = 0.01, *K* = 1, *t* = 10, *ω* = 0.2.

**Fig. 24:**
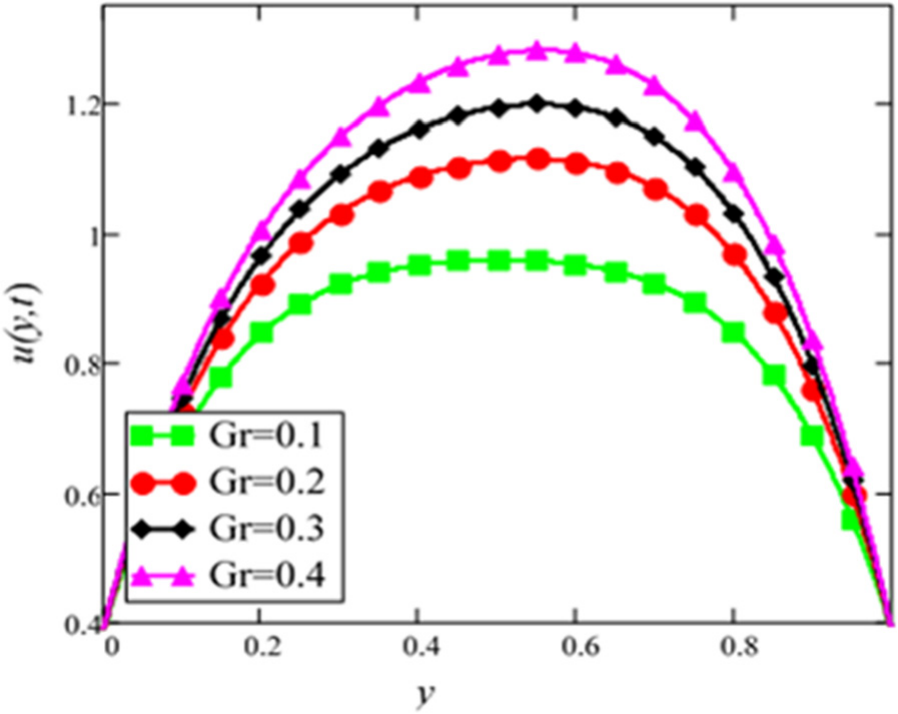
Velocity profile for different values of *Gr* in EG-based nanofluid when *N* = 0.1, *φ* = 0.04, *λ* = 0.01, *M* = 1, *K* = 1, *t* = 10, *ω* = 0.2.

**Fig. 25:**
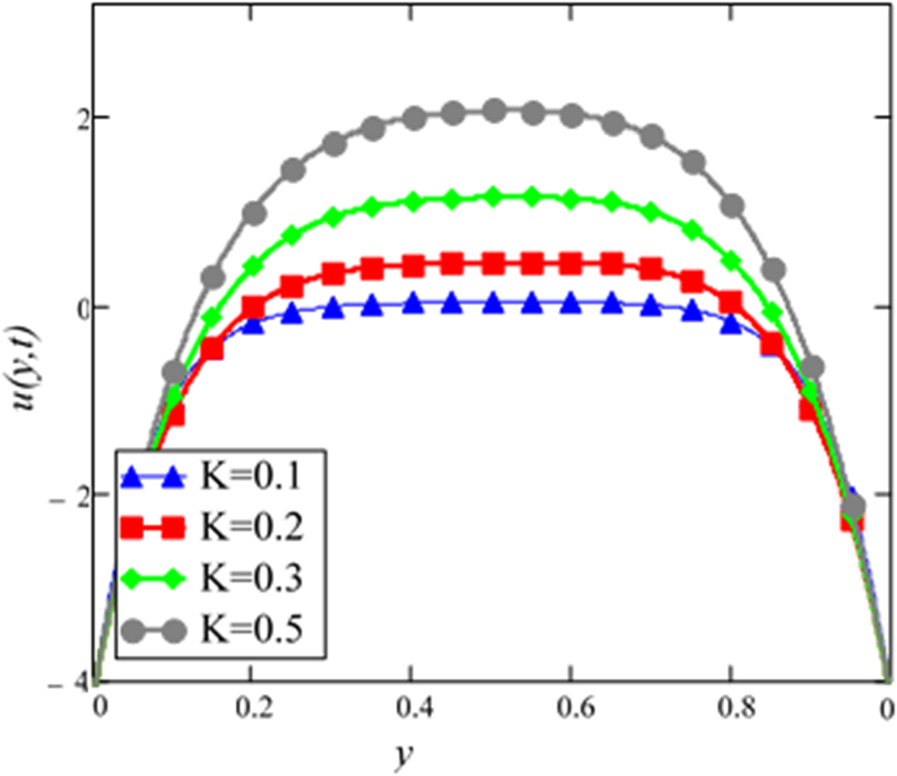
Velocity profile for different values of *K* in EG-based nanofluid when *N* = 0.1, *φ* = 0.04, *λ* = 0.01, *M* = 1, *t* = 10, *ω* = 0.2.

**Fig. 26:**
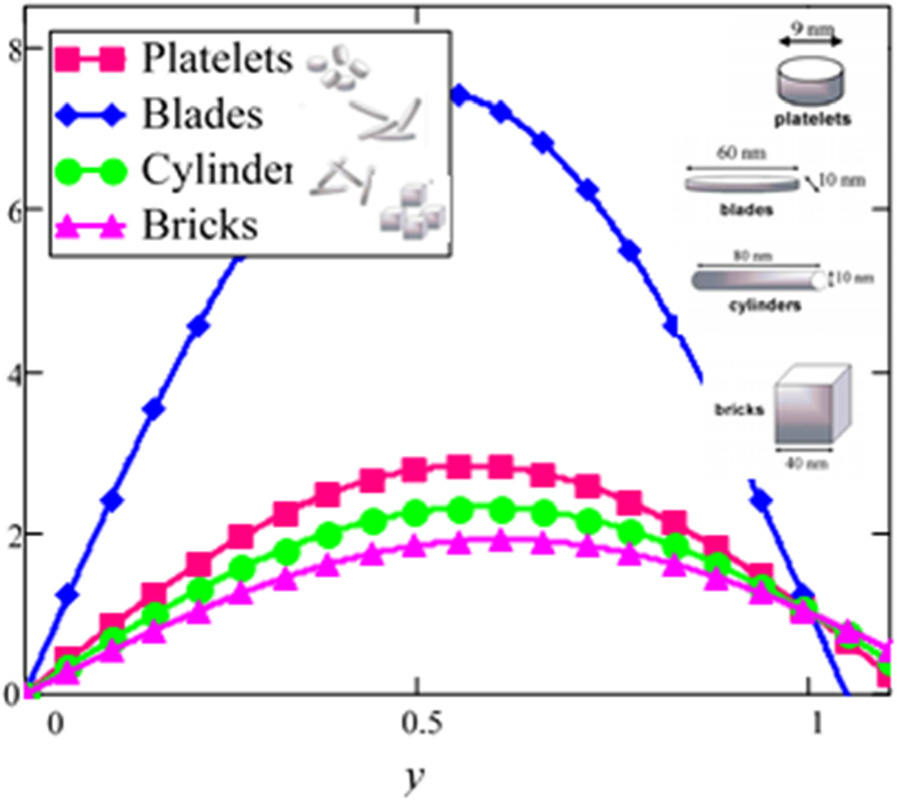
Temperature profile of different shapes of *Al*_2_ *O*_3_ nanoparticles in EG-based nanofluid when *N* = 1.5, *t* = 1.

**Fig. 27:**
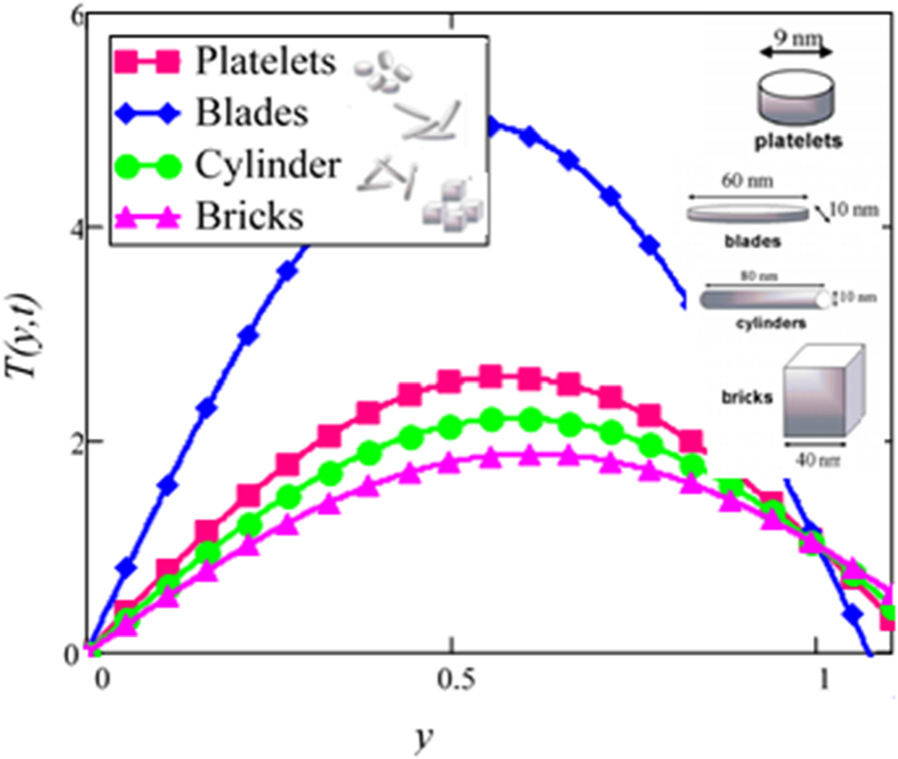
Temperature profile of different shapes of *Al*_2_ *O*_3_ nanoparticles in water-based nanofluid when *N* = 1.5, *t* = 1.

**Fig. 28:**
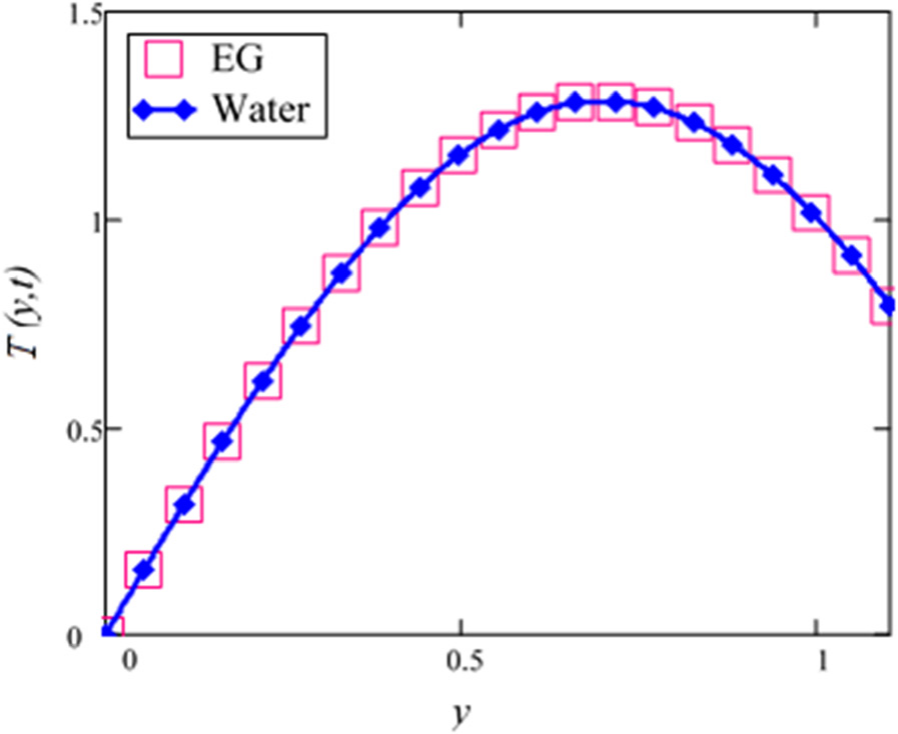
Temperature comparison graph of EG and water-based nanofluid when *N* = 1.5, *t* = 1.

**Fig. 29:**
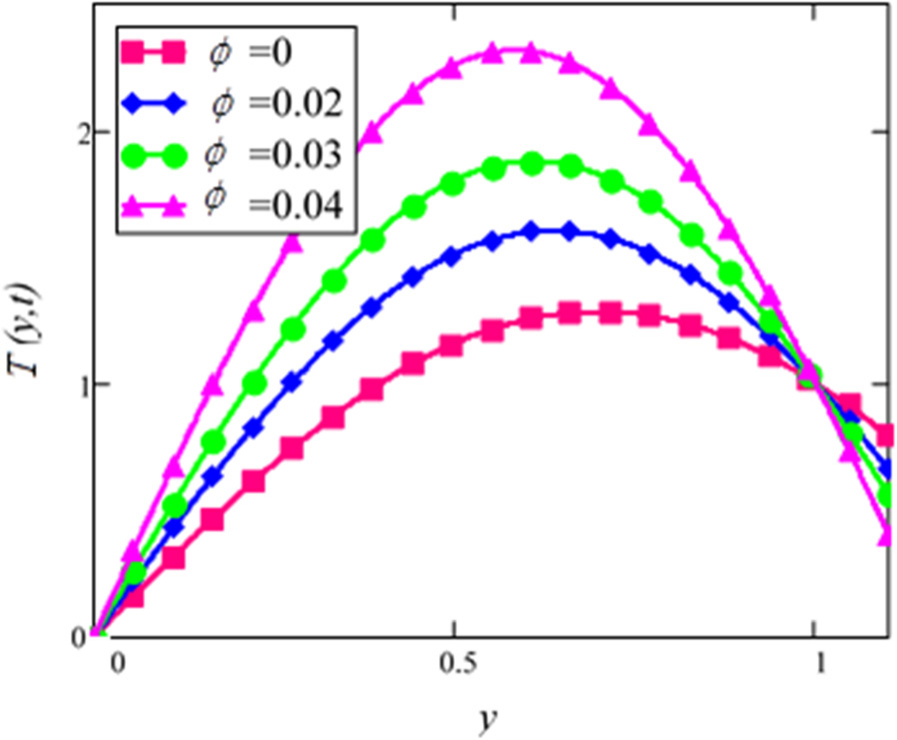
Temperature profile for different *∅* in EG based nanofluid when *N* = 1.5, *t* = 1.

**Fig. 30:**
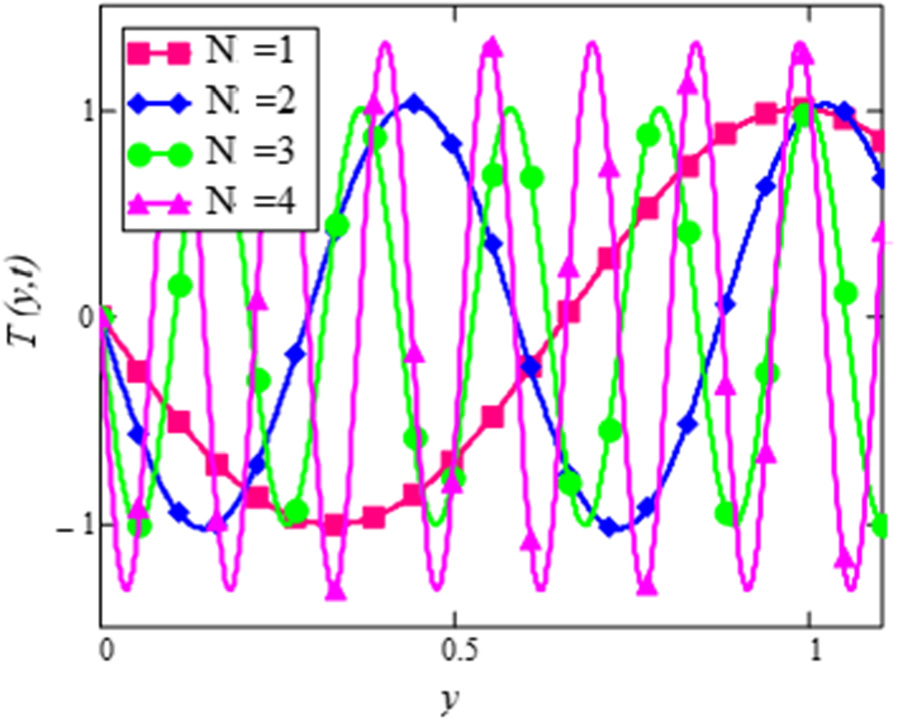
Temperature profile for different values of *N* in EG based nanofluid when *t* = 1.

Influence of radiation effect on heat transfer in mixed convection MHD flow of nanofluids inside a channel filled with saturated porous medium is studied. Based on the boundary conditions three different cases are discussed. Four different shapes of *Al*_2_*O*_3_ nanoparticles which are cylinder, platelet, brick and blade are dropped into conventional base fluid EG and water. The governing partial differential equations with imposed boundary conditions are solved for analytic solutions using perturbation technique. Expressions of velocity and temperature are obtained on the basis of Hamilton and Crosser model [28]. The physics of the problem is studied using various graphs and discussed in details for embedded parameters. The constants *α* and *b* (called empirical shape factors) are chosen from Table 1, and numerical values of sphericity ψ are chosen from Table 2. It should be noted that *a* and *b* coefficients vary significantly with particle shape. Four different shapes of nanoparticles (platelet, blade, cylinder and brick) of equal volume fraction are used in the numerical computation as given in Table 3. Figures 1, 2, 3, 4, 5, 6, 7, 8, 9, 10, 11, 12, 13, 14, 15, 16, 17, 18, 19, 20, 21, 22, 23, 24, and 25 are sketched for velocity profiles whereas Figs. 26, 27, 28, 29, and 30 are plotted for temperature profiles. Figures 1, 2, 3, 4, 5, 6, 7, 8, and 9, are plotted for the case when flow is inside a channel with stationary walls, Figs. 10, 11, 12, 13, 14, 15, 16, and 17 are sketched for the flow situation inside a channel with oscillating upper wall and Figs. 18, 19, 20, 21, 22, 23, 24, and 25 are drawn when both walls of the channel are executing the same oscillating motion.

The influence of different shapes of *Al*_2_*O*_3_ nanoparticles on the velocity of EG-based nanofluids is shown in Fig. 1. It can be seen from this figure that the blade shape *Al*_2_*O*_3_ nanoparticles has the highest velocity followed by brick, platelet and cylinder shapes nanoparticles. The influence of the shapes on the velocity of nanofluids is due to the strong dependence of viscosity on particle shapes for the volume fraction *ϕ* < 0.1. The present results show that the elongated shape nanoparticles like cylinder and platelet have the highest viscosities as compared to square shape nanoparticles like brick and blade. The obtained results agree well with the experimental results predicted by Timofeeva et al. [16]. A very small deviation is observed in the present study, where the cylinder shape nanoparticles has the highest viscosity, whereas from the experimental results reported by Timofeeva et al. [16] the platelet has the highest viscosity. Timofeeva et al. [16] had compared their results with Hamilton and Crosser model [28] and found that their results are identical with Hamilton and Crosser model [28]. In the present work, we have used Hamilton and Crosser model [28] and found that our analytical results also match with the experimental results of Timofeeva at el. [16].

Figure 2 is plotted to examine the effect of different shapes of *Al*_2_*O*_3_ nanoparticle on the velocity of water nanofluids. It is clearly seen that the cylinder shape *Al*_2_*O*_3_ nanoparticles has the lowest velocity followed by platelet, brick and blade. Thus, according to Hamilton and Crosser model [28], suspension of elongated and thin shape particles (high shape factor *n*) should have higher thermal conductivities, if the ratio of *k*_*nf*_/*k*_*f*_ is greater than 100. It is also mentioned by Colla et al. [32] that the thermal conductivity and viscosity increase with the increase of particle concentration due to which velocity decreases. Therefore, the cylinder shape *Al*_2_*O*_3_ nanoparticles has the highest thermal conductivity followed by platelet, brick and blade. Timofeeva et al. [16] described the reason that when the sphericity of nanoparticles is below 0.6, the negative contribution of heat flow resistance at the solid–liquid interface increases much faster than the particle shape contribution. Thus, the overall thermal conductivity of suspension start decreasing below sphericity of 0.6 but it is increasing in case of Hamilton and Crosser model [28] due to the only contribution of particle shape parameter *n*. Furthermore, flow in this research is single phase, therefore the negative contribution of heat flow resistance is neglected. Timofeeva et al. [16] used the model ${k}_{nf}/{k}_f=1+\left({c}_k^{shape}+{c}_k^{surface}\right)\;\phi$, for finding the thermal conductivity of nanoparticles. According to this model, ${c}_k^{shape}$ and ${c}_k^{surface}$ coefficients reflecting contributions to the effective thermal conductivity due to particle shape (positive effect) and due to surface resistance (negative effect) respectively. Particle shape coefficient ${c}_k^{shape}$ was calculated by Hamilton and Crosser equation.

A comparison of EG-based nanofluid with water-based nanofluid is made in Fig. 3. It is found that the velocity of water-based nanofluid is greater than the velocity of EG-based nanofluid. The viscosity and thermal conductivity of EG and water base nanofluids are also predicted by Hamilton and Crosser model [28] for the same ϕ. This result shows that EG based nanofluid has greater viscosity and thermal conductivity compared to water based nanofluid.

The effect of different nanoparticles on the velocity of nanofluids is presented in Fig. 4. From this figure, it is noted that cylinder shape *Al*_2_*O*_3_ nanofluid has the highest velocity followed by *Fe*_3_*O*_4_, *TiO*_2_, *Cu* and silver nanofluids. This shows that cylinder shape silver nanofluids has the highest viscosity and thermal conductivity compared to *Cu TiO*_2_, iron oxide *Fe*_3_*O*_4_ and *Al*_2_*O*_3_ nanofluids. One can see from this result that cylinder shape silver nanofluid has better quality fluids compared to magnetite cylinder shape *Fe*_3_*O*_4_ nanofluid. This result is supported by Hamilton and Crosser model [28] that the viscosity and thermal conductivity of nanofluid are also affected by nanoparticles *φ* i.e. the viscosity and thermal conductivity increase with the increase of *φ*, therefore, velocity decreases with the increase of *φ*. This figure further shows that viscosity of *Al*_2_*O*_3_ nanofluid at *φ* is less than 0.1 increases nonlinearly with nanoparticles concentration. This result is found identical with the experimental result reported by Colla et al. [32].

Different *φ* of cylinder shape *Al*_2_*O*_3_ nanoparticles on the velocity of cylinder shape *Al*_2_*O*_3_ nanofluid is shown in Fig. 5. It is clear from this figure that with the increase of *φ* velocity of nanofluids is decreased. This is due to the reason that the fluid becomes more viscous with the increase of *φ* which leads to decrease the velocity of nanofluids. The thermal conductivity of nanofluids also increase with the increase of *φ*. Experimentally by Colla et al. [32] also reported this behaviour. Results for different values of radiation parameter *N* are presented in Fig. 6. It is found that velocity increases with the increase of *N*. This result agrees well with the result obtained by Makinde and Mhone [31]. Physically, this means that with the increase of *N*, increases the amount of heat energy transfers to the fluid.

The graphical results of velocity for different values of magnetic parameter *M* are shown in Fig. 7. With increasing *M*, velocity of the nanofluid decreases. Increasing transverse magnetic field on the electrically conducting fluid gives rise to a resistive type force called Lorentz force which is similar to drag force and upon increasing the value of *M*, increases the drag force which has the tendency to slow down the fluid velocity. The drag force is maximum near the channel walls and minimum in the middle of the channel. Therefore, velocity is maximum in the middle of the channel and minimum at the boundaries.

The velocity profile for different value of Grashof number *Gr* is plotted in Fig. 8. It is found that an increase in *Gr*, leads to an increase in the velocity. Increase of *Gr*, increases temperature gradient which leads to an increase in the buoyancy force. Therefore, velocity increase occurs with *Gr*, is due to the enhancement of buoyancy force. Figure 9 is prepared for permeability parameter *K*. It is found that velocity increases with increasing *K* due to less friction force. More exactly, increasing *K* reduce fluid friction with channel wall and velocity enhances.

In the second case, Figs. 10, 11, 12, 13, 14, 15, 16, and 17 are plotted for the flow situation when the upper wall is oscillating and the lower wall is at rest. For the last case, when both boundaries are oscillating, Figs. 18, 19, 20, 22, 23, 24, and 25 are plotted. From all these graphs (Figs. 10, 11, 12, 13, 14, 15, 16, 17, 18, 19, 20, 21, 22, 23, 24, and 25), we found that they are qualitatively similar but different quantitatively to Figs. 1, 2, 3, 4, 5, 6, 7,8, and 9.

The effect of different particle shapes on the temperature of the nanofluid is shown in Figs. 26 and 27. The temperature in the present work is different for different shapes due to different viscosity and thermal conductivity of different shapes of nanoparticles. It should be noted that the effect of thermal conductivity increases with the increase of temperature but the viscosity decreases with the increase of temperature. It is clear that elongated shape of nanoparticles like cylinder and platelet have minimum temperature because of the greater viscosity and thermal conductivity whereas blade has the highest temperature due to least viscosity and thermal conductivity. The brick shape is lowest in temperature range, although, it has low viscosity. This is due to the shear thinning behavior with temperature. Further, cylinder shape also show shear thinning behavior but the effect is less prominent here. All the other shapes like platelet and blade show Newtonian behavior and independence of viscosity on shear rate. This shear thinning behavior is also studied experimentally by Timofeeva et al. [16].

Figure 28 shows a comparison of water and EG-based nanofluids. It is evaluated that both are temperature dependent and the variation is observed at the same rate for both fluids. This means that the effect of temperature on the thermal conductivity and viscosity of different based nanofluids occur at the same rate. Figure 29 is plotted in order to see the effect of *φ* on the temperature of the EG-based nanofluid. It is observed that with the increase of *φ* temperature of the fluid increases due to the shear thinning behavior. The viscosity of cylinder shape nanoparticles show shear thinning behavior at the highest concentration. This was also experimentally shown by Timofeeva et al. [16]. The graphical results of temperature for different values of radiation parameter *N* are shown in Fig. 30. It is clear from this figure that temperature of the cylinder shape nanoparticles in EG-based nanofluid get more sinusoidal with the increase of *N*. The increasing *N* means cooler or dense fluid or decrease the effect of energy transport to the fluid. The cylinder shape nanofluid has temperature dependent viscosity due to shear thinning behavior.

## Conclusions

In this paper, the effects of radiative heat transfer in mixed convection MHD flow of a different shapes of *Al*_2_*O*_3_ in ethylene glycol and water-based nanofluids in a channel filled with saturated porous medium are analyzed. The channel with non-uniform walls temperature is taken in a vertical direction under the influence of a transverse magnetic field. The governing partial differential equations are solved by perturbation technique for three different flow situations and analytic solutions are obtained. The influence of the different shapes of nanoparticles namely platelet, blade, cylinder and brick of equal volume on the velocity and temperature of nanofluids is determined with different results. Elongated particles like cylinder and platelet result in higher viscosity at the same volume fraction due to structural limitation of rotational and transitional Brownian motion. The shear thinning behavior of cylinder and blade shape of nanoparticles is also studied in this work. Viscosities and thermal conductivities of nanofluids are shown depending on particle shapes, volume fraction and base fluid of nanoparticles. The concluded remarks are as follow:

- The velocity of nanofluid decrease with the increase of volume fraction of nanoparticles due to increase of viscosity and thermal conductivity.
- Velocity of EG-based nanofluid is concluded lower than water-based nanofluid because the viscosity of base fluid effect the Brownian motion of the nanoparticles.
- Elongated particles like cylinder and platelet shapes have lower velocity as compared to blade and brick shapes of nanoparticles due to higher viscosity.
- The velocity of the nanofluid decrease with the increase of magnetic parameter due to increase of the drag force which has the tendency to slow down the motion of the fluid.
- The velocity of the nanofluid also increase with the increase of thermal Grashof number. Increasing of thermal Grashof number, temperature gradient increases which leads to increase the buoyancy force.
